# Patterns of Species Richness and Turnover for the South American Rodent Fauna

**DOI:** 10.1371/journal.pone.0151895

**Published:** 2016-03-21

**Authors:** Renan Maestri, Bruce D. Patterson

**Affiliations:** 1 Programa de Pós-Graduação em Ecologia, Universidade Federal do Rio Grande do Sul, Porto Alegre, RS, 91501–970, Brazil; 2 Integrative Research Center, Field Museum of Natural History, Chicago, Illinois, 60605, United States of America; Universidad de Chile, CHILE

## Abstract

Understanding the spatial distribution of species sheds light on the group’s biogeographical history, offers clues to the drivers of diversity, and helps to guide conservation strategies. Here, we compile geographic range information for South America’s diverse rodents, whose 14 families comprise ~50% of the continent’s mammalian species. The South American rodent fauna is dominated by independent and temporally staggered radiations of caviomorph and sigmodontine groups. We mapped species richness and turnover of all rodents and the principal clades to identify the main predictors of diversity patterns. Species richness was highest in the Andes, with a secondary hotspot in Atlantic Forest and some regions of considerable richness in Amazonia. Differences in richness were evident between the caviomorphs and sigmodontines, the former showing the greatest richness in tropical forests whereas the latter show—and largely determine—the all-rodent pattern. Elevation was the main predictor of sigmodontine richness, whereas temperature was the principal variable correlated with richness of caviomorphs. Across clades, species turnover was highest along the Andes and was best explained by elevational relief. In South America, the effects of the familiar latitudinal gradient in species richness are mixed with a strong longitudinal effect, triggered by the importance of elevation and the position of the Andes. Both latitudinal and elevational effects help explain the complicated distribution of rodent diversity across the continent. The continent’s restricted-range species—those seemingly most vulnerable to localized disturbance—are mostly distributed along the Andes and in Atlantic Forest, with the greatest concentration in Ecuador. Both the Andes and Atlantic Forest are known hotspots for other faunal and floral components. Contrasting patterns of the older caviomorph and younger sigmodontine radiations underscore the interplay of both historical and ecological factors in determining present-day diversity patterns.

## Introduction

A central question in studies of biodiversity concerns how species richness is distributed in space and where it varies and changes the most. By studying aggregate species distributions, macroecological studies can access hidden patterns and help to reveal the main factors explaining these patterns. Diversity in this context can be roughly distinguished in two components: alpha and beta [1]. Alpha diversity is simply the number of species present at a single site or its *species richness*; beta diversity, or here, *species turnover*, concerns changes in species composition among sites [2, 3]. Both components are important to understand how diversity is distributed across space and reflect the group’s biogeographic history as well as the ecological opportunities and challenges it has encountered over the course of its diversification. Additionally, both species richness and turnover provide critical information for conservation planning, identifying areas that should be conservation priorities [4].

One of the oldest and most general patterns of species richness is the latitudinal gradient of species richness [5–7]. Several hypotheses have been proposed to explain this richness pattern [8]. Among the most targeted in animal studies are the species-energy and the contemporary climate hypotheses. The species-energy hypothesis states that as the energy available in an ecosystem increases, it can therefore house more species [9, 10]. The species-energy hypothesis has (at least) three versions: the “productivity” and the “ambient-energy” hypotheses, which depend on whether energy influences richness through alimentary resources or thermoregulatory tolerances, respectively [8], and the “evolutionary speed” hypothesis, which relates energy with higher evolutionary rates in tropics ([5, 11] but see Bromham & Cardillo [12]). The contemporary climate hypothesis argues that climate-related features, including its stability, seasonality, and variability, act to shape patterns of diversity [13, 14] and promote the emergence of the latitudinal diversity pattern [5]. Furthermore, researchers are increasingly noting the effects of topographic complexity (i.e. variation in topography) on both richness [15] and turnover [2], although rises in diversity with increases in topographical complexity has long been appreciated [16]. Consequently, measures of climatic and topographic variables are likely to jointly affect diversity patterns over large spatial scales (e.g., [17]). Whereas variation in species richness is comparatively well studied, patterns and causes for variation in species turnover across large spatial scales is still poorly known [2].

South America offers a special case for studies of macroecology. The continent spans 65 degrees of latitude, including the Equator, and presents a dizzying range of tropical, temperate, and even subantarctic habitats. It has been isolated for most of the last 65 million years, almost as an island, with episodic connections for faunal exchanges with other parts of the world [18]. And it is home to the Andes Mountains, stretching 7000 km along the continent’s western margins, the longest continental mountain chain on Earth. These features have combined to generate the world’s richest vertebrate faunas [19] and floras [20]. Paradoxically, the challenges of revising and mapping its hyper-diverse faunas and floras have limited macroecological studies in South America to a few relatively well-studied groups at coarse taxonomic scales (e.g., birds: [8], mammals: [2, 21, 22], angiosperms: [23]).

Rodents comprise more than half of all Neotropical mammal species [24], and South America is home to about a quarter of all the world’s rodent species. Most are either “caviomorphs” (relatives of African mole-rats and Old World porcupines) or “sigmodontines” (a Neotropical radiation of the muroid family Cricetidae). Caviomorph ancestors arrived in South America during the Eocene (~50 Ma) via transoceanic dispersal from Africa [25] and the group underwent extensive diversifications in the Oligocene and Miocene [26]. Although many lineages are now extinct, nearly 250 species and 10 families range across the continent [27]. On the other hand, sigmodontine rodents (Cricetidae: Sigmodontinae) arrived in South America during the Miocene (~ 8 Ma, well before final closure of the Panamanian seaway), via island-hopping or transoceanic dispersal from North America [28–30]. Sigmodontines have radiated into 86 genera and nearly 400 species over this short time period [31]. Including squirrels, pocket mice, harvest mice and other groups, nearly 650 rodent species occur on the continent [27], exploiting fossorial, terrestrial, cursorial, arboreal, and semi-aquatic niches occupied by various mammal groups on other continents [32]. Caviomorphs and sigmodontines thus comprise the two principal monophyletic lineages of rodents in South America [33], with sharply contrasting histories of colonization of the continent [34]. Inside each radiation, phylogenetic analyses have established well-supported monophyletic lineages (i.e. clades) that are formally recognized and named. The older divergences among caviomorph lineages are recognized by placing their divisions into distinct superfamilies (Octodontoidea, Cavioidea, Chinchilloidea, and Erethizontidae [26]), whereas the younger sigmodontine lineages are recognized at the tribal level, grouping related genera within the subfamily Sigmodontinae (e.g. Oryzomyini, Akodontini, Thomasomyini, and Phyllotini [29, 35]).

An earlier analysis of rodent diversity in South America was based on distributions maintained by IUCN [22], which were produced in workshops during 2006 and 2007. Results pointed to four regions of high richness (the Andean yungas, western Amazonia, Atlantic Forest, and the Guianas) and to a modest concentration of threatened species in north-central Peru [22]. To date, no study has mapped the richness of the major clades of South American rodents (but see [26], which was also based on IUCN range maps) or explored their species turnover patterns. Recently, the taxonomy and geographical distribution of all South American rodents was comprehensively reviewed and revised by taxonomic experts [27]. This new revision permits more accurate analyses of rodent diversity and offers potentially new insights into their biogeography and conservation.

Here, we compiled the range maps of 653 species of rodents according to their distributions as given in Patton et al. [27]. We investigated patterns of species richness and turnover of all South American rodents and the two main clades (caviomorphs and sigmodontines), as well as their components (superfamilies and principal tribes, respectively). Although richness patterns of all mammals in South America have been addressed [36–39], studies are lacking for the major clades of South American rodents, and for the turnover patterns of these clades. We also assessed the distributions of restricted-range species, the quarter with the smallest ranges [40]. We used multiple regressions to evaluate which abiotic predictors might better explain species richness and turnover for these taxonomic groupings.

## Materials and Methods

### Data acquisition

Contributors to *The Mammals of South America*, *Vol*. *2*. *Rodents* [27] revised both the taxonomy and spatial distribution of each species of rodent occurring in South America. This was the most comprehensive revision of taxonomy since [41] and of their geographic ranges since [42]. We used the maps presented in the book to generate a digital image of the map for each species. Range maps of each species were then digitized to create *.shp files using the GSC South America 1969 projection and ArcMap ver. 9.2 software.

The range maps were then mapped onto a grid of 0.5° by 0.5° cells (~ 55 km at the Equator) which was pruned to cover the South American continent. A matrix of presence/absence of each species in each cell was created: species were considered present in a cell if their range occupied at least 50% of the cell. Based on this matrix, we defined the species richness of each cell by summing all the species occurring in it. Species turnover was calculated for each cell as the mean of the beta-diversity values between a focal cell and each of its eight adjacent cells [2]. The metric used to calculate species turnover follows the framework proposed by Baselga [3], where the turnover and nestedness components of beta diversity are decomposed. The spatial turnover component, used in this study, is calculated as a Simpson-based dissimilarity index (βSIM): min(*b*,*c*)/*a*+min(*b*,*c*), where *a* is the number of species common to both cells, *b* is the number of species exclusive to the focal cell, and *c* is the number of species exclusive to the adjacent cell. We chose βSIM because it is less sensitive to differences in species richness among cells [1].

Species turnover was quantified in R software [43], using the packages betapart [44] and CommEcol (package in development by Adriano S. Melo, available at: http://commecol.r-forge.r-project.org/). Because turnover values present a left-skewed distribution, we applied a square-root transformation of these values, which showed a normal distribution. Richness calculations and the diversity maps were constructed in SAM software (Spatial Analysis in Macroecology; [45]). All images generated were based on maps obtained from open sources (OpenStreetMap, free available at: http://www.openstreetmap.org/).

### Environmental correlates

We extracted four environmental variables from the Bioclim database [46] to use as predictors of species richness and turnover: 1) Elevation; 2) Mean temperature; 3) Mean precipitation; and 4) Seasonality in temperature. Temperature is the variable most closely associated with the energy hypothesis [5], elevation sought to capture topographic effects [15], and precipitation and seasonality are productivity- and climate-related features [47]. We chose these variables because they are commonly used in analyses of diversity patterns; studies with mammals have shown them to be correlated with both richness (e.g., [14, 15, 48]) and turnover (e.g., [2, 49]). We used values of the original variables in richness tests, on a cell-by-cell basis. However, the environmental variables were modified for correlations with species turnover: here, we employed mean differences of the values in the focal cell from its eight adjacent cells (see [2]). This approach sought to capture neighborhood differences in environmental metrics, and do so at the same spatial scale as the turnover metric itself. Hereafter, we refer to these variables in the text adding the suffix “.dif”, to distinguish them from the original variables used in richness tests.

We tested multicollinearity among the predictors by examining the variance inflation factor (VIF). Heuristically, values lower than 10 are taken as evidence of low collinearity between predictors [50]. VIF for our four predictor variables always returned a value lower than 7 in all partial regression tests (see Statistical Analyses), so we opted to use all four variables as predictors. Variables were extracted for each cell using SAM [45]. Mean differences in predictor values of the focal cell from its adjacent cells were calculated using the *select*.*window* function of the CommEcol package in R [43]. VIF tests were performed with the function *vif*.*cca* of the package vegan [51].

### Statistical analyses

We used multiple regressions to assess the effect of environmental variables on both species richness and turnover, as well as spatial terms to include spatial autocorrelation in the models. Spatial autocorrelation was first evaluated using Moran’s I correlograms [52], for both species richness and turnover, for all rodents and for each clade in separate (Moran’s correlograms appear in S1 Appendix). We then calculated principal coordinates of neighborhood matrices (PCNM) by performing a principal coordinate analysis (PCoA) on the truncated distance matrix connecting all sites [53]. Truncation distance was defined under a minimum-spanning-tree criterion [54]. Eigenvectors from this PCoA were then selected under the criterion of minimizing Moran’s I residuals, and the selected eigenvectors were used in the regressions to correct estimated effects of the predictors, taking into account their spatial autocorrelation [55]. These eigenvectors (spatial filters) represent different spatial gradients, where those with higher eigenvalues characterize broad-scale spatial gradients, whereas eigenvectors with small eigenvalues characterize small scale gradients [53]. Each partial regression was carried out on species richness or turnover using a single environmental variable as predictor at a time, controlling for the effect of spatial filters and for the effects of the other environmental variables. In this way, the independent effect of each variable could be assessed. A model-selection technique based on information theory [56] was used as an alternative to partial regression in order to assess simultaneously the importance of all predictors included in the analysis. The Akaike information criterion (AIC) was used in model selection, and the relative importance of predictors in the best models were ranked by their standardized regression coefficients. PCNM extraction and partial regressions were performed in the R environment [43] with the package vegan [51], via *pcnm* and *rda* functions; model selection based on AIC was conducted with the package MuMIn [57]. The relationships between diversity metrics with latitude and longitude were evaluated by simple Pearson’s correlations.

## Results

### Species richness

**Fig 1:**
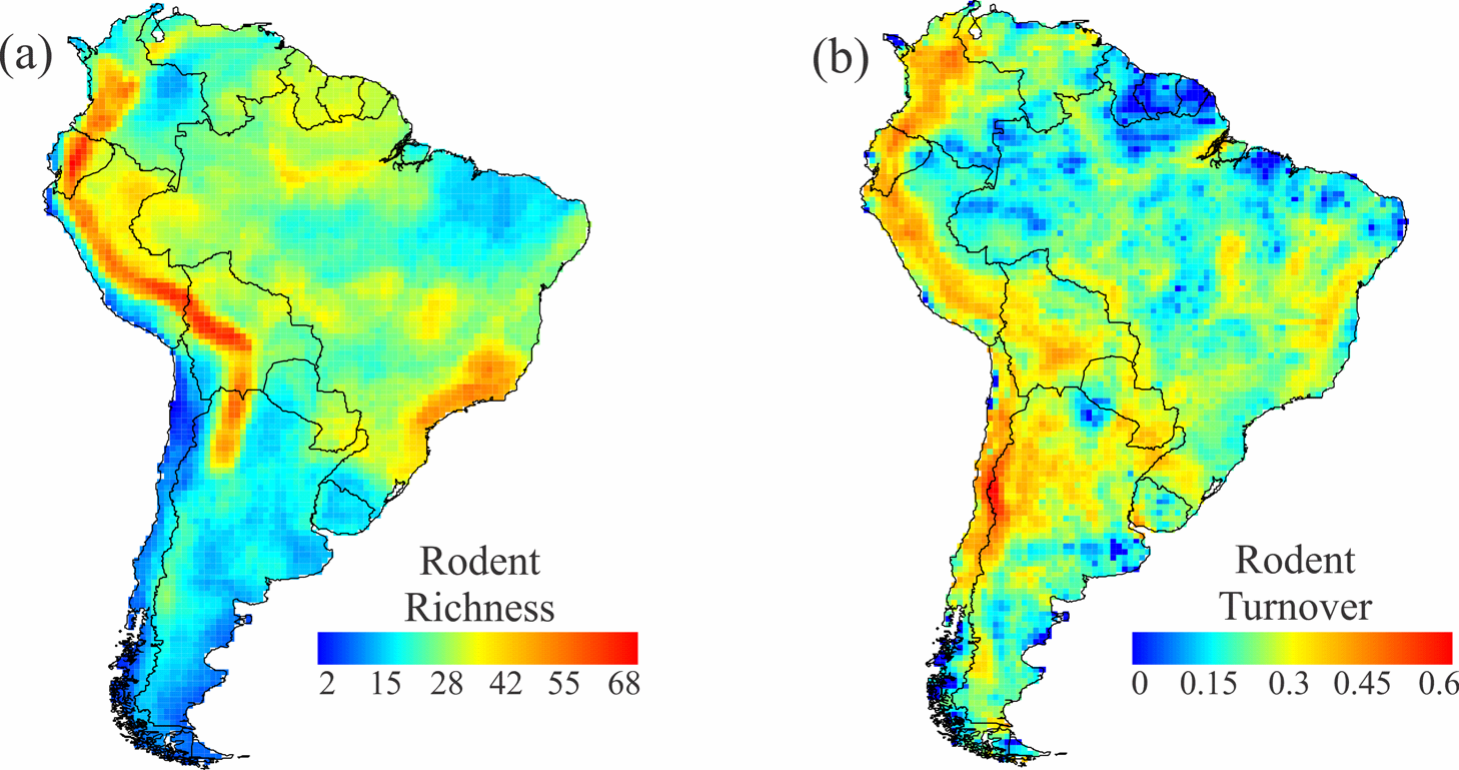
Rodent richness and turnover across South America. (a) Rodent richness, and (b) its turnover. Turnover was calculated as the average of the Simpson-dissimilarity index (βSIM—[3]) between a focal cell and each of its eight neighboring cells.

**Fig 2:**
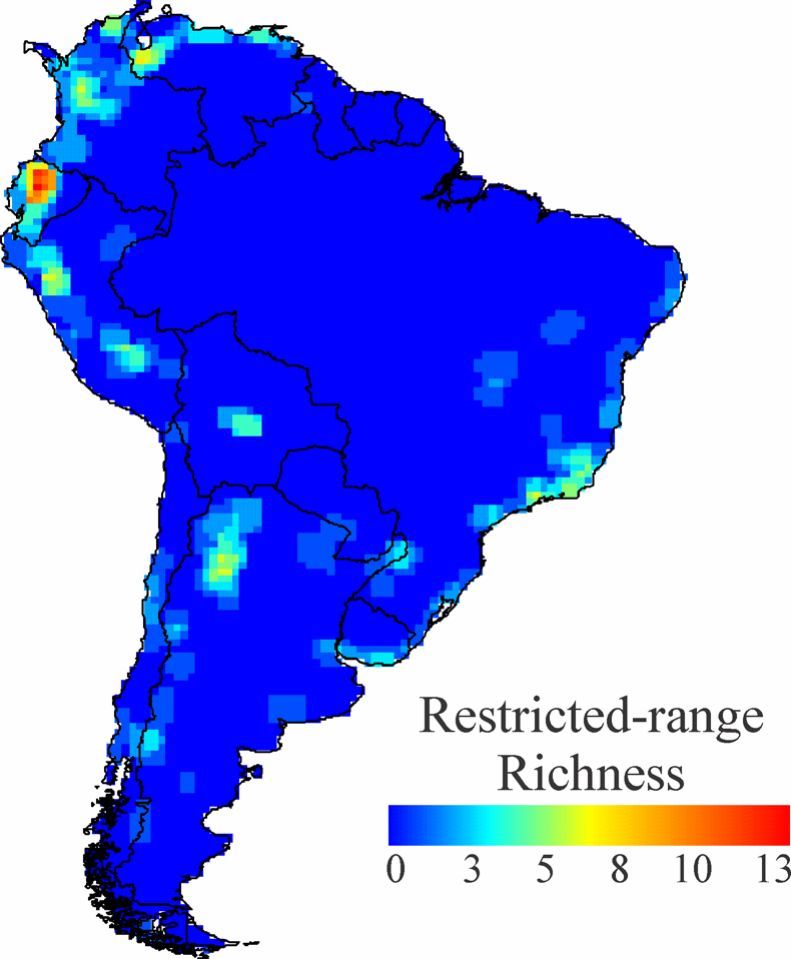
Richness of restricted-range species. Richness of the 25% of species with the smallest ranges.

The overall pattern of rodent diversity is depicted in Fig 1A. High richness is concentrated along the Andes, from Colombia to northern Argentina, with a second hotspot in the Brazilian Atlantic Forest. Other regions, such as western and eastern Amazonia, also support substantial richness. Restricted-range rodents (the quartile of species with the smallest ranges; see S2 Appendix) are mostly distributed in the Andes, from Mérida (Venezuela) to Tucuman (Argentina), with a great concentration in Ecuador, as well as in the Atlantic Forest of Brazil and Argentina (Fig 2).

**Fig 3:**
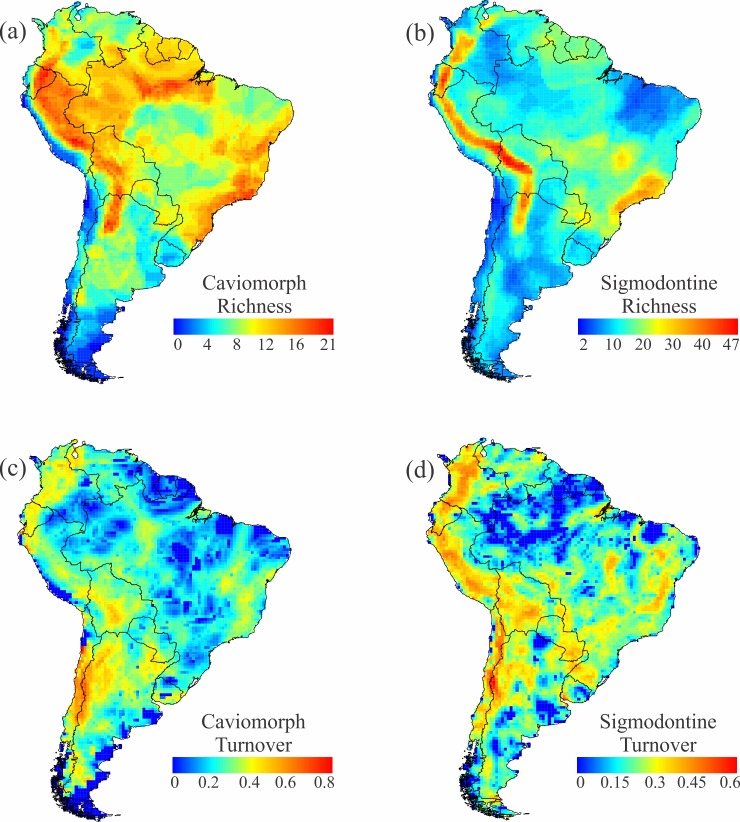
Richness and turnover of rodent clades across South America. (a) Caviomorph richness; (b) Sigmodontine richness; (c) Caviomorph turnover; (d) Sigmodontine turnover. Turnover was calculated as the average of the Simpson-dissimilarity index (βSIM—[3]) between a focal cell and each of its eight neighboring cells.

Species richness of caviomorphs is high along the Andes, through much of Amazonia and Atlantic Forest, and in some regions of central and northeastern Brazil (Fig 3A). Sigmodontines are rich all along the tropical Andes, with lesser peaks in Atlantic Forest and in the Cerrado (Fig 3B). It is noteworthy that these richness patterns are relative and ignore absolute differences in richness between caviomorphs and sigmodontines; the latter are richer across virtually all of South America. The richness pattern of sigmodontines strongly influences the overall richness pattern, based on 14 families of rodents.

**Fig 4:**
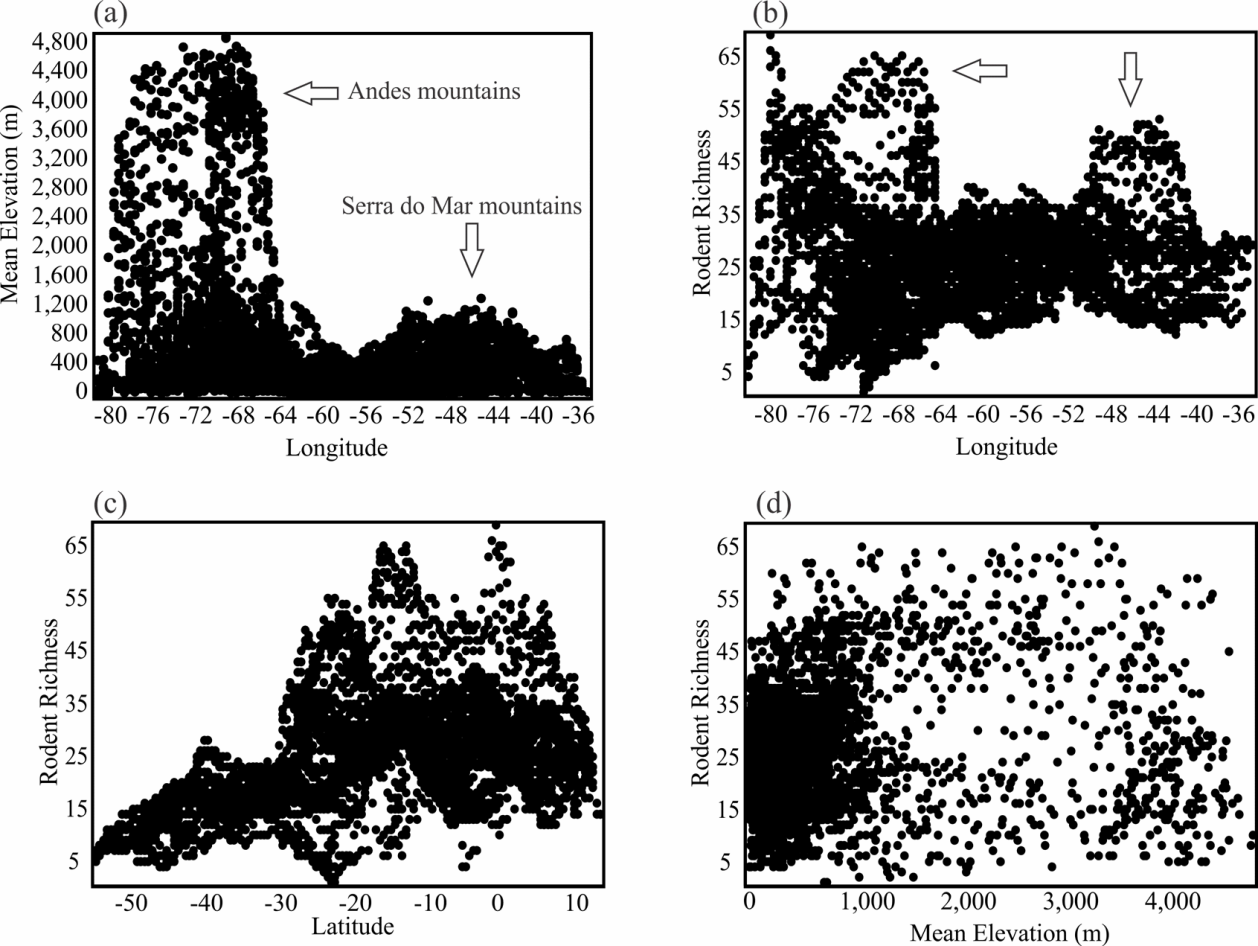
The relationship among rodent richness, latitude, longitude, and elevation. (a) The relationship between mean elevation (m) and longitude (r = -0.26), (b) rodent richness and longitude (r = 0.01), (c) rodent richness and latitude (r = 0.39), and (d) rodent richness and mean elevation (m) (r = 0.14).

**Fig 5:**
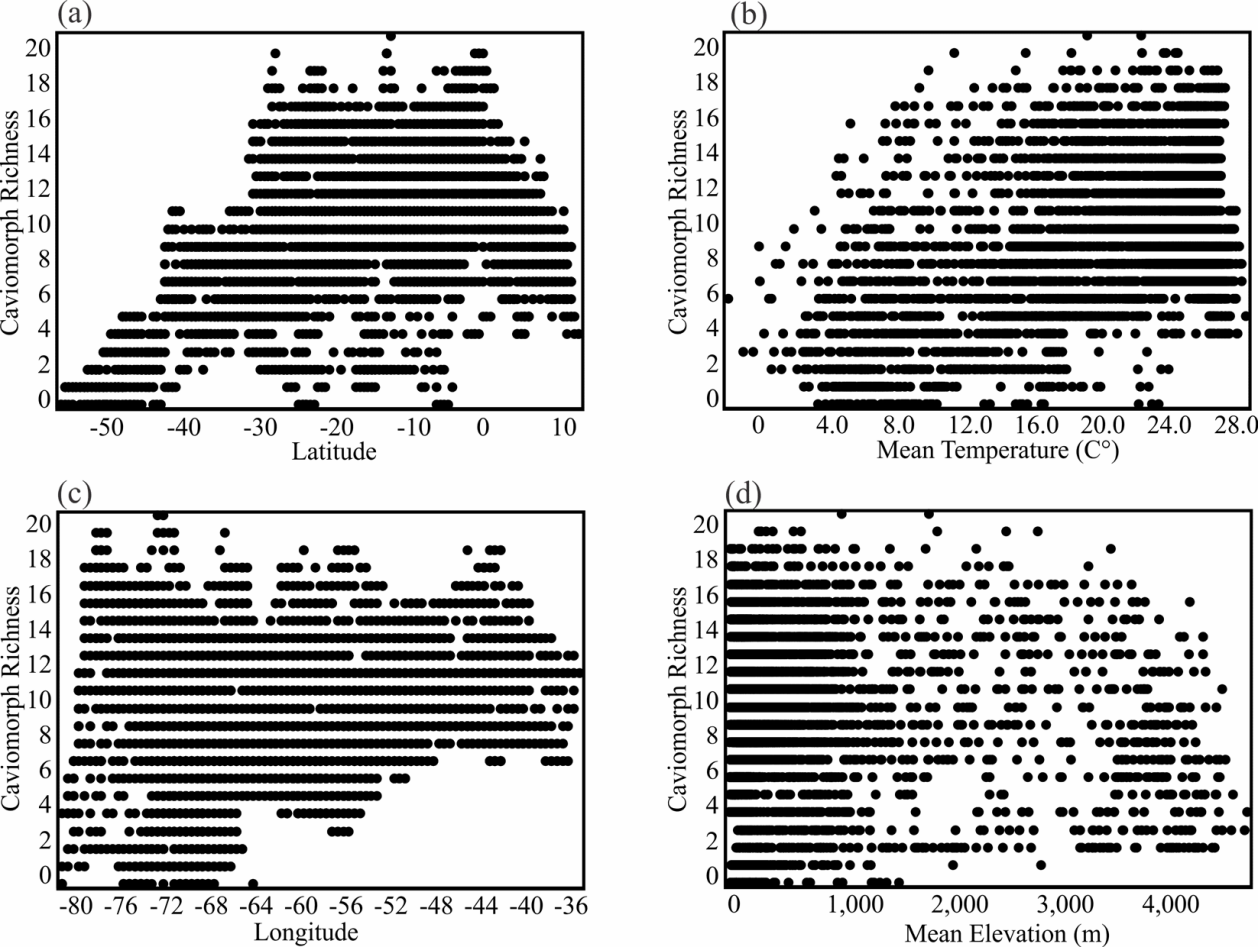
The relationship among caviomorph richness, latitude, longitude, temperature and elevation. (a) The relationship between caviomorph richness and latitude (r = 0.50), (b) caviomorph richness and mean temperature (C°) (r = 0.50), (c) caviomorph richness and longitude (r = 0.17), and (d) caviomorph richness and mean elevation (m) (r = -0.07).

**Fig 6:**
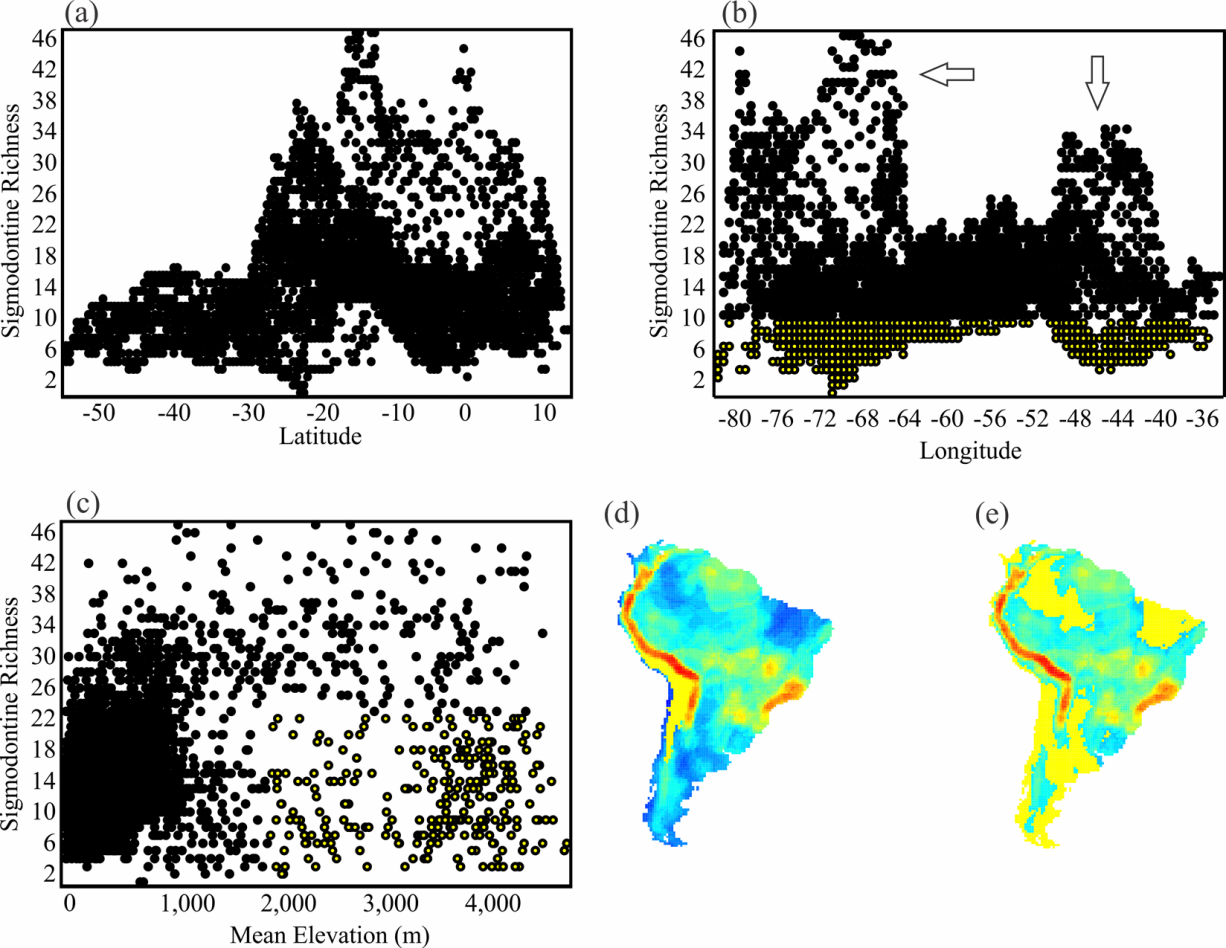
The relationship among sigmodontine richness, latitude, longitude, and elevation. (a) The relationship between sigmodontine richness and latitude (r = 0.14), (b) sigmodontine richness and longitude (r = 0.01), (c) sigmodontine richness and mean elevation (m) (r = 0.31). Cells highlighted in yellow in plots (b) and (c) are depicted in yellow in the corresponding maps (e) and (d), respectively.

Rodent richness is positively correlated with latitude (r = 0.39, Fig 4C). The pattern is strong for caviomorphs (r = 0.50, Fig 5A), and weaker for sigmodontines (r = 0.14, Fig 6A). Richness patterns are also influenced by elevation (rodents, r = 0.14; caviomorphs, r = -0.07; and sigmodontines, r = 0.31). This correlation and the presence of the Andes along the continent’s western margins mean that richness is also correlated with longitude. Relationships between elevation, longitude and species richness are shown in Fig 4. The plot of elevation on longitude (Fig 4A) shows the imprints of both the Andes in the west and the Serra do Mar in the east. The plot of rodent richness against longitude shows that peaks in elevation and species richness are largely coincident (Fig 4B). Nevertheless, there is considerable variation in richness across both longitude (Fig 4B) and elevation (Fig 4D), and neither variable explains much variation in species richness.

Caviomorph richness is instead highly associated with latitude and with temperature (r = 0.50, Fig 5B), and less influenced elevation (Fig 5D). Plots of caviomorph richness lack the imprint of Andes in their relationship with longitude (Fig 5C).

Sigmodontine patterns (Fig 6) strongly contribute to the rodent-wide patterns and show the same general associations. Despite scatter, there are obvious latitudinal, elevation and longitudinal relationships.

**Fig 7:**
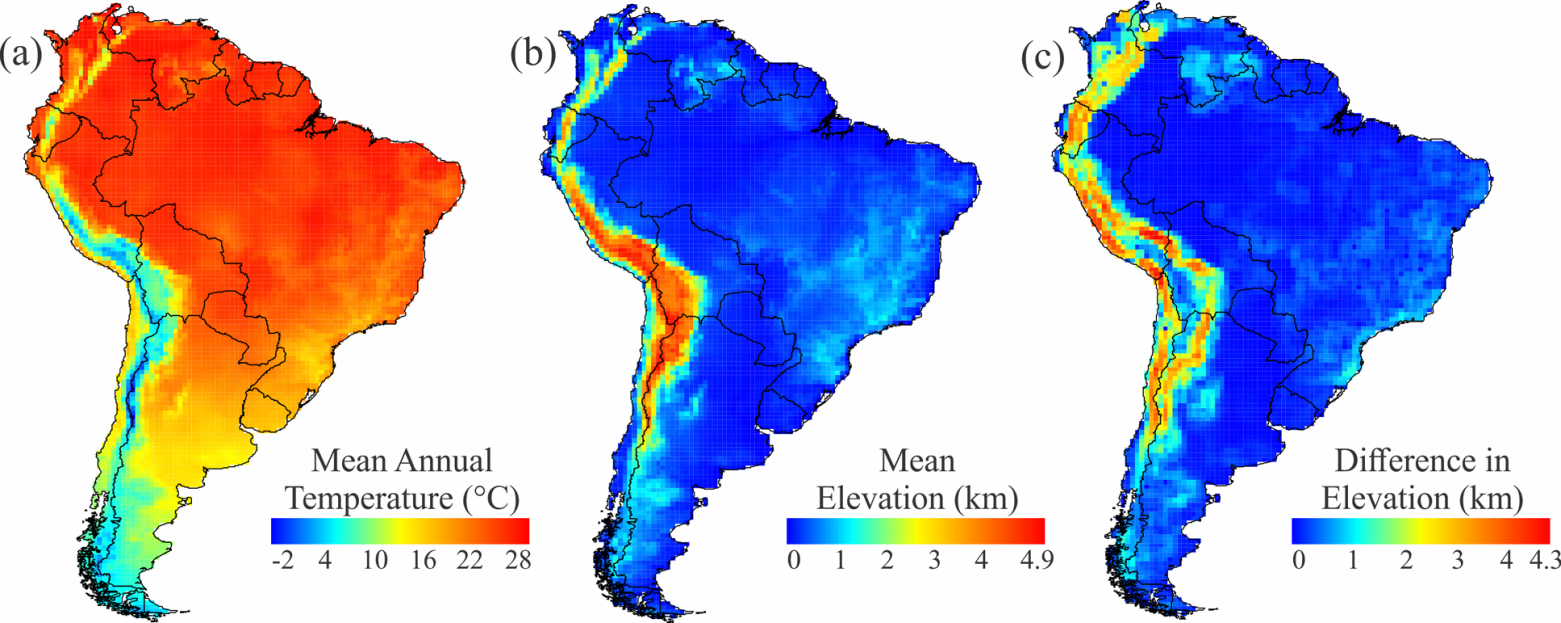
Predictors of rodent richness and turnover. (a) Mean Annual Temperature, one of the major predictors of rodent richness; (b) Mean elevation, one of the major predictors of rodent richness; (c) Differences in elevation between a focal cell and its neighbors, the main predictor of species turnover.

There was significant spatial autocorrelation in all response variables (i.e. richness and turnover for all rodents, caviomorphs and sigmodontines), with similar patterns of positive spatial autocorrelation at smaller scales and mostly negative autocorrelation at larger ones (see S1 Appendix). In general, both partial regressions and model-selection procedures returned similar results concerning the importance of each predictor in explaining diversity patterns (Tables 1 and 2). The main predictors of species richness for all rodents were mean elevation and mean temperature (Tables 1 and 2); positively associated with rodent richness. Caviomorph richness was mainly influenced by temperature, whereas sigmodontine richness was more strongly affected by elevation. Precipitation and seasonality in temperature had smaller influences on overall richness, but contributed modestly to models of caviomorph and sigmodontine richness (Table 2). Maps of temperature (Fig 7A), elevation (Fig 7B), and topographic complexity (Fig 7C) for South America are shown in Fig 7.

**Table 1:** Partial regression analysis of richness and turnover of rodents in South America.

Predictors	Richness					
	All Rodents		Caviomorphs		Sigmodontines	
	R^2^	F	R^2^	F	R^2^	F
Global model	0.095	379.34	0.166	723.07	0.069	215.90
Elevation	**0.055**	**882.54**	0.043	762.27	**0.049**	**606.01**
Temperature	**0.044**	**714.00**	**0.107**	**1859.7**	0.011	142.26
Precipitation	0.024	382.72	0.011	191.88	0.017	213.32
Seasonality	0.010	169.02	0.018	324.80	0.007	94.412
Predictor	Turnover					
	All Rodents		Caviomorphs		Sigmodontines	
	R^2^	F	R^2^	F	R^2^	F
Global model	0.110	319.90	0.060	147.74	0.087	232.65
Elevation.dif	**0.044**	**513.66**	**0.021**	**215.30**	**0.032**	**346.73**
Temperature.dif	0.002	26.401	0.001	16.867	0.001	19.52
Precipitation.dif	0.0009	11.446	0.0002	2.768	0.0001	0.06
Seasonality.dif	0.002	25.230	0.0008	8.779	0.002	24.56

The values of R^2^ and F are provided for the global model and for each predictor after accounting for the others. Spatial autocorrelation was controlled by using spatial filters as a condition variable in all models (see Methods). Most important variables appear in bold.

**Table 2:** Multiple regression models for richness and turnover of rodents in South America.

Species richness							
	Elev	Temp	Prec	Seas	R^2^	AICc	AIC_c_ *w*_*i*_
All Rodents	0.521	0.553	0.266	0.343	0.61	401.3	0.99
Caviomorphs	0.463	0.835	0.105		0.64	281.2	0.99
Sigmodontines	0.49	0.28	0.225	0.177	0.50	365.7	0.99
Species turnover							
	Elev.dif	Temp.dif	Prec.dif	Seas.dif	R^2^	AICc	AIC_c_ *w*_*i*_
All Rodents	0.384	0.08	-0.037	-0.057	0.46	-163.4	0.98
Caviomorphs	0.27	0.069	-0.02	-0.036	0.37	-105.1	0.57
Sigmodontines	0.33	0.072		-0.059	0.41	-134.1	0.73

Only the models with lowest AICc are shown. The standardized regression coefficients of the predictors included in each model are provided, along with the R^2^, AICc and the AIC weighting of each model (AIC_c_ w_i_). Correction for spatial autocorrelation was made by including spatial filters as a fixed variable in all models.

**Fig 8:**
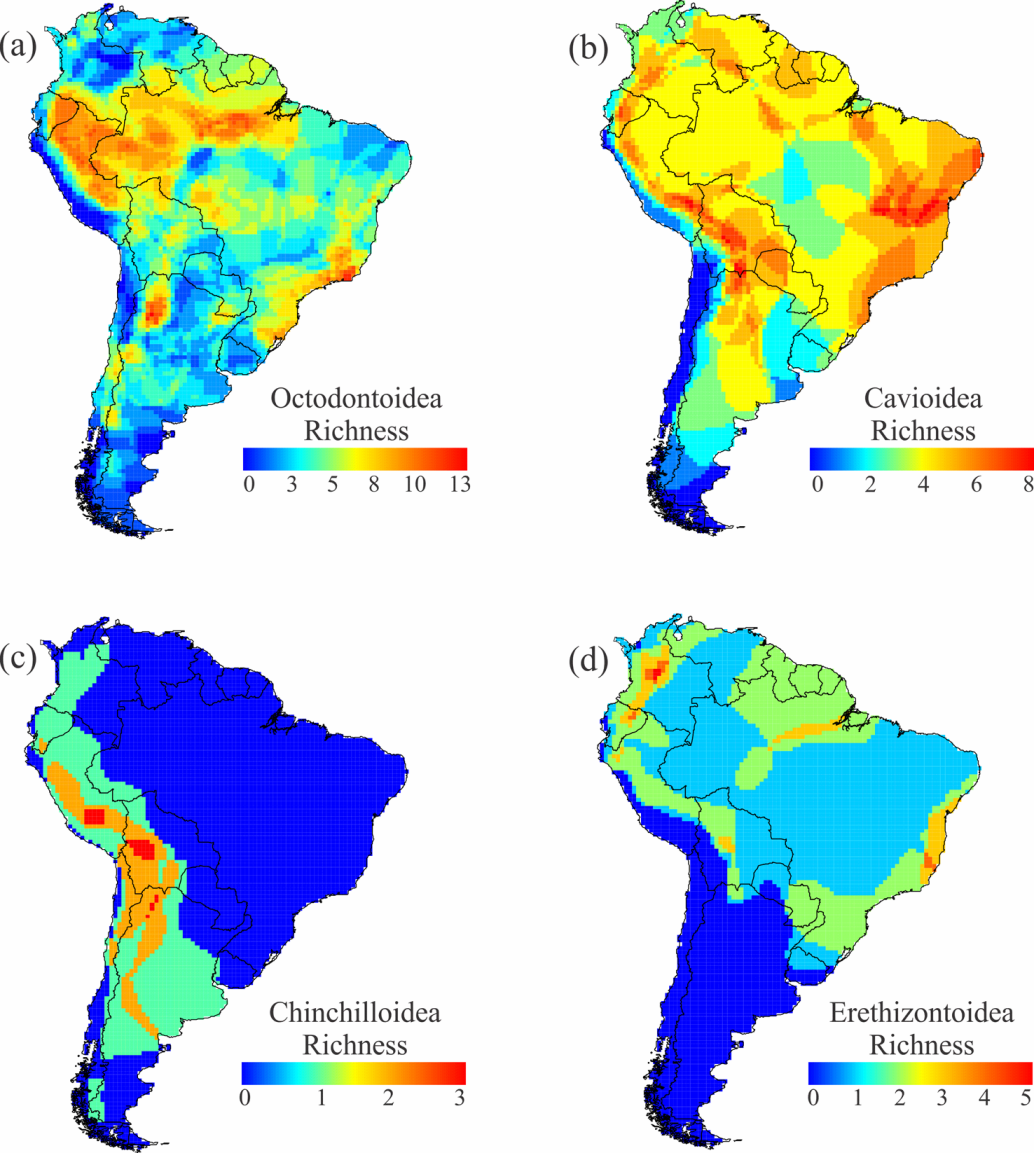
Richness of the four superfamilies of caviomorphs in South America. Richness of (a) Octodontoidea, (b) Cavioidea, (c) Chinchilloidea, and (d) Erethizontoidea in the South American portions of their ranges.

Richness of caviomorph superfamilies in South America is shown in Fig 8. The richest superfamily, Octodontoidea (spiny rats and allies, 182 species), has diversity hotspots in Amazonia and the Atlantic forest, as well as in northern Argentina (Fig 8A). Cavioidea (guinea pigs and allies, 34 species) are rich in the central Andes and the Caatinga (Fig 8B). Living species of Chinchilloidea (chinchillas and pacaranas, 8 species) are restricted to western South America, mainly in the Andes (Fig 8C). Lastly, Erethizontidae (New World porcupines, 14 species) have disjunct centers of richness, with peaks in the Atlantic Forest of Brazil and the northern Andes of Colombia (Fig 8D).

**Fig 9:**
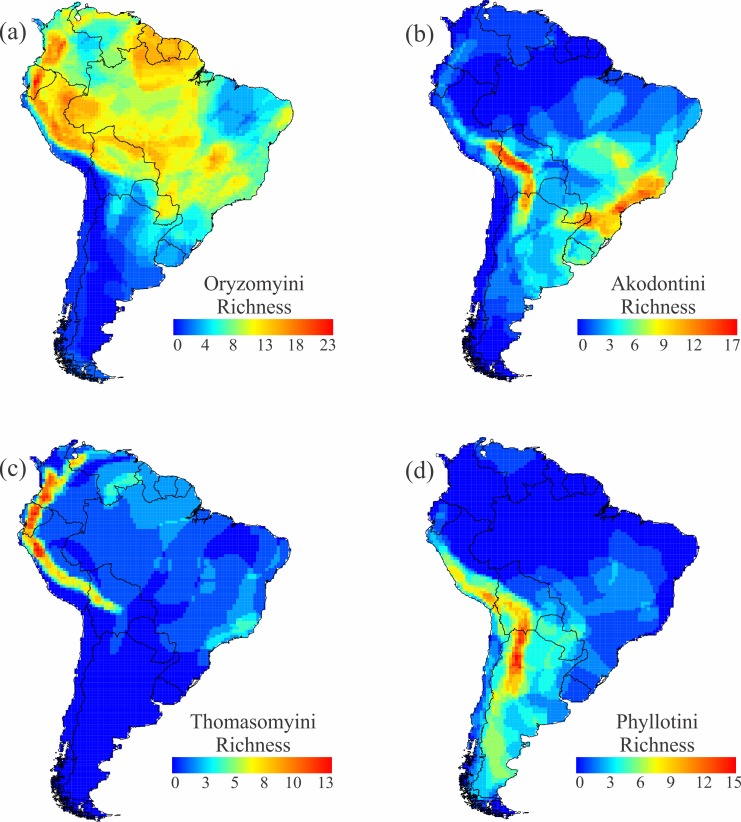
Richness of the main tribes of sigmodontines. Richness of (a) Oryzomyini, (b) Akodontini, (c) Thomasomyini, and (d) Phyllotini.

The richness patterns of the main tribes of sigmodontines appear to be largely complementary to one another (Fig 9). Species of the largest tribe, Oryzomyini (rice rats, 121 species), are richest in northern South America, with hotspots of diversity in the northern and central Andes, western Amazonia, the Guianas, and the Cerrado (Fig 9A). In contrast, species of the Akodontini (field mice, 85 species) are concentrated in two hotspots, in the central Andes and the Atlantic forest (Fig 9B). Species of Thomasomyini (Thomas’ mice, 74 species) are strongly concentrated in the northern and central Andes, where they overlap with oryzomyines but complement the Andean distributions of akodontines and phyllotines (Fig 9C). Species of Phyllotini (leaf-eared mice, 51 species) overlap with akodontines in the central Andes, but are richer toward the southern tip of the continent (Fig 9D).

### Species turnover

Rodent turnover was generally highest all along the Andes, with the region of greatest turnover in the southern Andes (Fig 1B). Other regions, including the Atlantic Forest-Pampas and Atlantic Forest-Cerrado ecotones, also presented moderate species turnover. The Guianas and most of Amazonia are characterized by low species turnover. This general pattern was evident for both caviomorphs and sigmodontines, exaggerated in the latter by their higher species richness (Fig 3C and 3D). Spatial differences between these groups include little caviomorph turnover across the Peruvian Andes, where turnover of sigmodontines is high, and far greater turnover of sigmodontines along the margins of the Brazilian Plateau, where Amazonia, Cerrado, Caatinga, and Atlantic Forest all abut one another. Both groups show strong turnover between interior portions of the Atlantic Forest and the more open formations to the west and south (Fig 3C and 3D).

The best predictor of species turnover (all rodents, caviomorphs, and sigmodontines) was elevation (Tables 1 and 2). Elevational relief is high along both slopes of the Andes mountain chain (Fig 4B), where it is associated with elevated species turnover in rodents (Figs 1B, 3C and 3D). The turnover component shows a correlation of 0.52 with elevation.dif, -0.24 with longitude, and -0.14 with latitude.

## Discussion

The pattern of high species richness and turnover being associated with complex topographies has long been recognized and holds for many taxa [15, 16]. Many species of both Phyllostomidae (bats) and Cricetidae (rodents) reach their distributional range limits of species along the mountain chain [58]; this concentration brings various lowland and upland faunas into close proximity. Here we demonstrate that the Andes host both the highest species richness and species turnover of rodents in South America. Elevational measures offer the best explanations for both richness and turnover patterns for all rodents (Tables 1 and 2). By establishing barriers to dispersal and isolating populations, thus leading to speciation [15], mountain ranges help generate a high richness and turnover [2,14,15].

The richness of rodents in general, and of sigmodontines in particular, is strongly affected by elevation, which is dominated by the Andes and introduces an indirect effect of longitude. The richness of caviomorphs, on the other hand, is positively associated with temperature, which correlates well with latitude. The turnover component is greatly affected by elevational relief, a pattern that holds across all clades (Tables 1 and 2). Thus, latitudinal effects on species richness of South American rodents are mixed with elevational effects, and consequently by longitude, while species turnover is more closely associated with elevation and less with latitude.

The species-energy hypothesis, therefore, fails to explain diversity patterns of rodents in South America. High-energy environments support great diversity, especially of caviomorphs, but low-energy mountains habitats can harbor an even greater diversity of rodents. Different mechanisms appear to explain these patterns. High-energy environments may contribute to increases in diversity though ecological mechanisms (e.g. productivity, evolutionary speed), as hypothesized. But elevation per se, by disrupting species ranges, may contribute to allopatric speciation and vicariant ecological replacements, even where overall productivity is low (cf. Fig 1B). Differences between high- and low-elevation sites in diversity would be diminished if considered in terms of biomass, given the much larger average size of caviomorphs [32]. Disentangling the various mechanisms by which energy can act is beyond the scope of this paper.

The positive association of species richness with elevation is not universal though. In fact, for most groups, the decrease in energy availability with increasing elevation diminishes the number of species (see [5, 59] for reviews). This is often not the case for mammals [15], as demonstrated here at a macroscale. The pattern of high richness associated with high elevations was not clear indeed, especially because some exceptionally arid regions of the western Andes (Fig 6C and 6E) support low species despite their elevational complexity. Such differences may explain why some local or regional studies detect diminishing richness of rodents at higher elevations (e.g., [59]).

Bats (Chiroptera) are the second-richest order of mammals in terms of species. Mammal-wide studies of diversity patterns demonstrate that bats have a strong influence on the latitudinal richness gradient [6, 60], and often exhibit the most pronounced latitudinal gradients [21, 61]. Although the latitudinal pattern also holds for non-volant taxa, rodents often do not follow this gradient [60]. We also recovered this pattern and identify elevational effects as a possible explanation.

The general rodent richness pattern is different from that presented in [22] using the IUCN database. That analysis reported higher species richness in Guianan forests, not evident in our analyses (Fig 1A), and lower richness in the Andes, especially to the south in Bolivia and northern Argentina. Nevertheless, these overall richness patterns are based on fundamentally different patterns shown by the continent’s principal rodent radiations (caviomorphs and sigmodontines).

The four caviomorph superfamilies all date to the Oligocene (>32 Ma; [26]) and each underwent substantial Cenozoic radiations in the absence of other rodents and various other groups [32]. In fact, more genera of Cavioidea, Chinchilloidea, and Erethizontoidea are known from the Miocene (23–5.3 Ma) than are extant in those groups today [62]. Most living genera of caviomorphs had already appeared by the end of the Miocene [26]. The caviomorph radiations can be considered mature and are obvious products of both speciation and extensive extinction. Although Chinchilloidea species are now limited to Andean and peri-Andean regions, fossils show that they were ubiquitous in the Miocene. The other superfamilies are generally diverse in the same regions (Fig 8): western Amazonia, along the Andes, and along the Atlantic coast of Brazil. The present-day diversity patterns of caviomorphs can offer only a weak signal of their historic diversification patterns (but see [63], for reconstructions based on their phylogenetic patterns).

On the other hand, the sigmodontine tribes and genera appeared only in the late Miocene and Pliocene, 6–2.5 Ma [29, 30], so that their radiations are far younger than the caviomorphs. Although the sigmodontines are distributed throughout the continent, each of the major tribes has diversity hotspots that are largely complementary to one another (Fig 9). The central and southern Andes constitute the chief exception, being a region of overlap where all four major tribes exhibit elevated richness. The central location of this region allows the juxtaposition of different regional faunas, and its topographic complexity allows these to occupy diverse habitats that are zoned by elevation. By interrupting and limiting distributions, topographic complexity promotes both higher species richness and turnover. The complementarity of tribal distributions is also evident: oryzomyines are the dominant sigmodontines in Amazonia and range well into mountainous regions in western Amazonia and the Guiana shield, but exhibit lower richness along Brazil’s Serra do Mar (Fig 9A). That same Atlantic Forest region houses a hotspot of akodontine richness, and this group is scarcely present in Amazonian forest (Fig 9B). These two rainforests are similar environmentally and share many widespread species [64]. Historical contingences are likely responsible for the geographically segregated but complementary diversity patterns of sigmodontines (see also [65]). Phylogenetic methods are now being applied to help resolve these relationships [35, 66].

The turnover pattern documented for all rodents are similar to that for all mammals depicted in Melo et al. [2]. Differences in elevation were the main predictor of turnover in their study, as in ours. South America has been called “The Rodent Continent” (R. S. Voss in [27]), and the dominance of rodents (~50% of all species) certainly contributes to these similarities between studies involving all mammals and those focusing solely on rodents. There are dramatic changes in rodent species composition along the Andes from one cell to another, both vertically and horizontally. Studies of widespread Andean forest birds have shown that their geographic distributions average 300 times longer than they are wide, following the ribbon-like distribution of suitable habitat along Andean slopes [67]. Flight allows these animals to cross the intervening river canyons that drain the Eastern Versant. But studies on rodents have shown that speciation often occurs by allopatric divergence in separate watersheds along the Andean versant [68, 69]; species may subsequently become closely juxtaposed via elevational zonation, producing both high richness and high turnover [70].

Elevation thus affects these distributions both historically, by limiting geographic ranges [58] and setting the stage for allopatric speciation, and ecologically, by creating a vertical succession of habitats suitable for a plethora of species [71]. The relative importance of historical or ecological components are apt to vary from place to place and across spatial scales.

Species with small geographic ranges are expected to be more vulnerable to habitat conversion and other localized anthropogenic threats [40]. Restricted-range species of rodents in this study occur mostly in the tropical Andes, especially Ecuador, as well as in the Atlantic Forest. These regions present elevated richness and turnover of rodents, and are characterized by substantial topographic relief that is dissected by river valleys. This spatial pattern was also documented for all terrestrial mammals [72], but it contrasts with recent proposals for rodents based on IUCN Redlist classifications. Using older IUCN distributions, [22] showed that vulnerable species were geographically scattered save for a small concentration in the Peruvian Andes. Because a number of the restricted-range species used in our analysis do not yet have IUCN classifications, conducting reviews of their status (and reassessing this discrepancy) should be a high conservation priority.

Our study demonstrated that a latitudinal gradient in species richness is coupled with an elevational gradient of great importance in explaining rodent richness and turnover in South America. This finding highlights the importance of the Andes in shaping diversity patterns in the continent, and points to the role of elevation in forging macroecological gradients for terrestrial mammals. Richness, and especially species turnover, are better associated with elevational effects than with latitudinal effects. Caviomorphs and sigmodontines showed different richness patterns, which underscores the importance of treat different evolutionary radiations separately. Future studies might investigate the influence of stochastic processes on richness, such as the mid-domain effect [48]. We hope the newly generated information will help to guide strategies for conserving the extraordinary diversity and vulnerability of faunas in the tropical Andes, the southern Andes, and the Atlantic forest.

## Supporting Information

S1 Appendix Moran’s I correlograms for rodent richness and turnover. (DOCX)

S2 Appendix Range sizes of rodent species. (DOCX)

## References

[1] Lennon JJ, Koleff P, Greenwood J, Gaston KJ. The geographical structure of British bird distributions: diversity, spatial turnover and scale. J Anim Ecol. 2001; 70(6):966–79.

[2] Melo AS, Rangel TFL, Diniz-Filho JAF. Environmental drivers of beta-diversity patterns in New World birds and mammals. Ecography. 2009; 32(2):226–36.

[3] Baselga A. Partitioning the turnover and nestedness components of beta diversity. Global Ecol Biogeogr Let. 2010; 19(1):134–43.

[4] McKnight MW, White PS, McDonald RI, Lamoreux JF, Sechrest W, Ridgely RS, et al Putting beta-diversity on the map: broad-scale congruence and coincidence in the extremes. PLoS Biol. 2007; 5(10):e272 17927449 10.1371/journal.pbio.0050272 PMC2001212

[5] Rohde K. Latitudinal gradients in species diversity: the search for the primary cause. Oikos. 1992:514–27.

[6] Kaufman DM. Diversity of New World mammals: universality of the latitudinal gradients of species and bauplans. J Mamm. 1995; 76(2):322–34.

[7] Rodríguez P, Arita HT. Beta diversity and latitude in North American mammals: testing the hypothesis of covariation. Ecography. 2004; 27(5):547–56.

[8] Hawkins BA, Porter EE, Diniz-Filho JAF. Productivity and history as predictors of the latitudinal diversity gradient of terrestrial birds. Ecology. 2003; 84(6):1608–23.

[9] Hutchinson GE. Homage to Santa Rosalia or why are there so many kinds of animals? Am Nat. 1959; 93:145–59.

[10] Wright DH. Species-energy theory: an extension of species-area theory. Oikos. 1983; 41(3):496–506.

[11] Tamma K, Ramakrishnan U. Higher speciation and lower extinction rates influence mammal diversity gradients in Asia. BMC Evol Biol. 2015; 15(1):11. 25648944 10.1186/s12862-015-0289-1 PMC4333168

[12] Bromham L, Cardillo M. Testing the link between the latitudinal gradient in species richness and rates of molecular evolution. Journal of Evolutionary Biology. 2003; 16(2):200–7. 14635858 10.1046/j.1420-9101.2003.00526.x

[13] Rahbek C, Graves GR. Multiscale assessment of patterns of avian species richness. Proc Natl Acad Sci U S A. 2001; 98(8):4534–9. 11296292 10.1073/pnas.071034898 PMC31869

[14] Tognelli MF, Kelt DA. Analysis of determinants of mammalian species richness in South America using spatial autoregressive models. Ecography. 2004; 27(4):427–36.

[15] Badgley C. Tectonics, topography, and mammalian diversity. Ecography. 2010; 33(2):220–31.

[16] Simpson GG. Species density of North American recent mammals. Syst Zool. 1964:57–73.

[17] Jetz W, Fine PVA. Global gradients in vertebrate diversity predicted by historical area-productivity dynamics and contemporary environment. PLoS Biol. 2012; 10(3):e1001292 10.1371/journal.pbio.1001292 22479151 PMC3313913

[18] Patterson BD, Costa LP, editors. Bones, Clones, and Biomes: The history and geography of Recent Neotropical mammals. Chicago: University of Chicago Press; 2012.

[19] Jenkins CN, Pimm SL, Joppa LN. Global patterns of terrestrial vertebrate diversity and conservation. Proc Natl Acad Sci U S A. 2013; 110(28):E2602–E10. 10.1073/pnas.1302251110 23803854 PMC3710798

[20] Kier G, Kreft H, Lee TM, Jetz W, Ibisch PL, Nowicki C, et al A global assessment of endemism and species richness across island and mainland regions. Proceedings of the National Academy of Sciences. 2009; 106(23):9322–7. 10.1073/pnas.0810306106 PMC2685248 19470638

[21] Pereira MJR, Palmeirim JM. Latitudinal diversity gradients in New World bats: are they a consequence of niche conservatism? PLoS One. 2013; 8(7):e69245 10.1371/journal.pone.0069245 23935963 PMC3720615

[22] Amori G, Chiozza F, Patterson BD, Rondinini C, Schipper J, Luiselli L. Species richness and distribution of Neotropical rodents, with conservation implications. Mammalia. 2013; 77(1):1–19.

[23] Kerkhoff AJ, Moriarty PE, Weiser MD. The latitudinal species richness gradient in New World woody angiosperms is consistent with the tropical conservatism hypothesis. Proceedings of the National Academy of Sciences. 2014; 111(22):8125–30. 10.1073/pnas.1308932111 PMC4050539 24847062

[24] Patterson BD. Patterns and trends in the discovery of new Neotropical mammals. Divers Distrib. 2000; 6:145–51.

[25] Rowe DL, Dunn KA, Adkins RM, Honeycutt RL. Molecular clocks keep dispersal hypotheses afloat: evidence for trans-Atlantic rafting by rodents. J Biogeogr. 2010; 37(2):305–24. doi: j.1365-2699.2009.02190.x.

[26] Upham NS, Patterson BD. Evolution of caviomorph rodents: a complete phylogeny and timetree for living genera In: Vassallo AI, Antenucci D, editors. Biology of caviomorph rodents: diversity and evolution. Buenos Aires: SAREM Series A; 2015 p. 63–120

[27] Patton JL, Pardiñas UFJ, D'Elia G, editors. Mammals of South America, Vol. 2: Rodents. Chicago: University of Chicago Press; 2015.

[28] Steppan SJ, Adkins RM, Anderson J. Phylogeny and divergence-date estimates of rapid radiations in muroid rodents based on multiple nuclear genes. Syst Biol. 2004; 53(4):533–53. 15371245 10.1080/10635150490468701

[29] Parada A, Pardinas UFJ, Salazar-Bravo J, D'Elía G, Eduardo Palma R. Dating an impressive Neotropical radiation: Molecular time estimates for the Sigmodontinae (Rodentia) provide insights into its historical biogeography. Mol Phylogen Evol. 2013; 66:960–8. 10.1016/j.ympev.2012.12.001 23257216

[30] Vilela JF, Mello B, Voloch CM, Schrago CG. Sigmodontine rodents diversified in South America prior to the complete rise of the Panamanian Isthmus. Journal of Zoological Systematics and Evolutionary Research. 2013; 52(3):249–56. 10.1111/jzs.12057

[31] Lessa EP, Cook JA, D'Elía G, Opazo JC. Rodent diversity in South America: transitioning into the genomics era. Frontiers in Ecology and Evolution: Phylogenetics, Phylogenomics, and Systematics. 2014; 2(39):1–7. 10.3389/fevo.2014.00039

[32] Mares MA, Ojeda RA. Patterns of diversity and adaptation in South American hystricognath rodents In: Mares MA, Genoways HH, editors. Mammalian biology in South America. Pymatuning symposia in ecology Pittsburgh: Pymatuning Laboratory of Ecology, University of Pittsburgh; 1982 p. 393–432.

[33] Fabre PH, Hautier L, Dimitrov D, Douzery EJP. A glimpse on the pattern of rodent diversification: a phylogenetic approach. BMC Evol Biol. 2012; 12:88 10.1186/1471-2148-12-88 22697210 PMC3532383

[34] Patterson BD, Upham NS. A study in contrasts: two extensive Neotropical radiations. Frontiers in Ecology and Evolution. 2014; 2:44 10.3389/fevo.2014.00044

[35] Leite RN, Kolokotronis S-O, Almeida FC, Werneck FP, Rogers DS, Weksler M. In the wake of invasion: tracing the historical biogeography of the South American cricetid radiation (Rodentia, Sigmodontinae). PLoS One. 2014; 9(6):e100687 10.1371/journal.pone.0100687 24963664 PMC4071052

[36] Ojeda RA. Diversity and conservation of Neotropical mammals In: Levin SA, editor. Encyclopedia of Biodiversity, 2nd edition, Volume 2 Waltham, MA: Academic Press; 2013 p. 582–94.

[37] Ruggiero A. Latitudinal correlates of the sizes of mammalian geographic ranges in South America. J Biogeogr. 1994; 21:545–59.

[38] Ruggiero A, Kitzberger T. Environmental correlates of mammal species richness in South America: effects of spatial structure, taxonomy and geographic range. Ecography. 2004; 27(4):401–16.

[39] Ruggiero A, Lawton JH, Blackburn TM. The geographic ranges of mammalian species in South America: spatial patterns in environmental resistance and anisotropy. J Biogeogr. 1998; 25(6):1093–103.

[40] Terborgh J. Preservation of natural diversity: The problem of extinction-prone species. BioSci. 1974; 24:715–22.

[41] Musser GG, Carleton MD. Superfamily Muroidea In: Wilson DE, Reeder DAM, editors. Mammal species of the world: a taxonomic and geographic reference, 3rd ed. 2 Baltimore: Johns Hopkins University Press; 2005 p. 894–1531.

[42] IUCN. IUCN Redlist of Threatened Species, 2010.2: International Conservation Union; 2008 [12 Aug 2010]. Available from: http://www.iucnredlist.org/.

[43] R Core Team. R: a language and environment for statistical computing Vienna, Austria: R Foundation for Statistical Computing; 2015.

[44] Baselga A, Orme D, Villeger S, De Bortoli J, Leprieur F. betapart: Partitioning beta diversity into turnover and nestedness components. R package version 1.3. 2013.

[45] Rangel TFLVB, Diniz-Filho JAF, Bini LM. SAM: a comprehensive application for Spatial Analysis in Macroecology. Ecography. 2010; 33:46–50.

[46] Hijmans RJ, Cameron SE, Parra JL, Jones PG, Jarvis A. Very high resolution interpolated climate surfaces for global land areas. International Journal of Climatology. 2005; 25(15):1965–78.

[47] Begon M. Ecology: from individuals to ecosystems, 4th edition Oxford: Blackwell; 2006.

[48] Stevens RD. Gradients of bat diversity in Atlantic Forest of South America: environmental seasonality, sampling effort and spatial autocorrelation. Biotrop. 2013; 45(6):764–70.

[49] López‐González C, Presley SJ, Lozano A, Stevens RD, Higgins CL. Ecological biogeography of Mexican bats: the relative contributions of habitat heterogeneity, beta diversity, and environmental gradients to species richness and composition patterns. Ecography. 2015; 38(3):261–72.

[50] Gross J. Variance inflation factors. R News. 2003; 3:13–5.

[51] Oksanen J, Blanchet FG, Kindt R, Legendre P, Minchin PR, O’Hara RB, et al. vegan: community ecology package. R package version 2.3.1. http://CRAN.R-project.org/package-vegan; 2015.

[52] Sokal RR, Oden NL, Thomson BA. Local spatial autocorrelation in biological variables. Biol J Linn Soc. 1998; 65(1):41–61.

[53] Borcard D, Legendre P. All-scale spatial analysis of ecological data by means of principal coordinates of neighbour matrices. Ecological Modelling. 2002; 153(1):51–68.

[54] Rangel TFL, Diniz-Filho JAF, Bini LM. Towards an integrated computational tool for spatial analysis in macroecology and biogeography. Global Ecol Biogeogr Let. 2006; 15(4):321–7.

[55] Diniz-Filho JAF, Bini LM. Modelling geographical patterns in species richness using eigenvector-based spatial filters. Global Ecol Biogeogr Let. 2005; 14(2):177–85.

[56] Burnham KP, Anderson DR. Model selection and multimodel inference: a practical information-theoretic approach New York: Springer Science & Business Media; 2002.

[57] Bartoń K. MuMIn: multi-model inference. R package version 1.15.1. http://CRAN.R-project.org/package=MuMIn 2013.

[58] Patterson BD, Solari S, Velazco PM. The role of the Andes in the diversification and biogeography of Neotropical mammals In: Patterson BD, Costa LP, editors. Bones, Clones, and Biomes: The history and geography of Recent Neotropical mammals. Chicago: University of Chicago Press; 2012 p. 351–78.

[59] Willig MR, Presley SJ. Biodiversity and metacommunity structure of animals along altitudinal gradients in tropical montane forests. J Trop Ecol. In press: 10.1017/S0266467415000589

[60] Buckley LB, Davies TJ, Ackerly DD, Kraft NJB, Harrison SP, Anacker BL, et al Phylogeny, niche conservatism and the latitudinal diversity gradient in mammals. Proc R Soc Lond B. 2010; 277(1691):2131. 10.1098/rspb.2010.0179 PMC2880153 20335205

[61] Willig MR, Patterson BD, Stevens RD. Patterns of range size, richness, and body size in the Chiroptera In: Kunz TH, Fenton MB, editors. Bat ecology. Chicago: University of Chicago Press; 2003 p. 580–621.

[62] Vucetich MG, Arnal M, Deschamps CM, Pérez ME, Vieytes EC. A brief history of caviomorph rodents as told by the fossil record In: Vassallo AI, Antenucci D, editors. Biology of caviomorph rodents: diversity and evolution. Buenos Aires: SAREM Series A; 2015.

[63] Upham NS, Patterson BD. Diversification and biogeography of the Neotropical caviomorph lineage Octodontoidea (Rodentia: Hystricognathi). Mol Phylogen Evol. 2012; 63:417–29. 10.1016/j.ympev.2012.01.020 22327013

[64] Costa LP. The historical bridge between the Amazon and the Atlantic Forest of Brazil: a study of molecular phylogeography with small mammals. J Biogeogr. 2003; 30(1):71–86.

[65] Patterson BD. Contingency and determinism in mammalian biogeography: the role of history. J Mamm. 1999; 80:345–60.

[66] Parada A, D’Elía G, Palma RE. The influence of ecological and geographical context in the radiation of Neotropical sigmodontine rodents. BMC Evol Biol. 2015; 15(1):172. 26307442 10.1186/s12862-015-0440-z PMC4549906

[67] Graves GR. Linearity of geographic range and its possible effect on the population structure of Andean birds. Auk. 1988; 105:47–52.

[68] Patton JL, Myers P, Smith MF. Vicariant versus gradient models of diversification: the small mammal fauna of eastern Andean slopes of Peru In: Peters G, Hutterer R, editors. Biogeography and systematics in the tropics, Bonn, June 5–8 1989. Bonn: Alexander Koenig Zoological Research Institute and Zoological Museum; 1990 p. 355–71.

[69] Patton JL, Smith MF. mtDNA phylogeny of Andean mice: a test of diversification across ecological gradients. Evolution. 1992; 46(1):174–83. 28564953 10.1111/j.1558-5646.1992.tb01992.x

[70] Voss RS. A new species of *Thomasomys* (Rodentia: Muridae) from eastern Ecuador, with remarks on mammalian diversity and biogeography in the Cordillera Oriental. Amer Mus Novit. 2003; 3421:1–47.

[71] Terborgh J. Distribution on environmental gradients. Theory and a preliminary interpretation of distribution patterns in the avifauna of the Cordillera Vilcabamba, Peru. Ecology. 1971; 52:23–40.

[72] Schipper J, Chanson JS, Chiozza F, Cox NA, Hoffmann M, Katariya V, et al The status of the world's land and marine mammals: diversity, threat and knowledge. Science. 2008; 322:225–30. 10.1126/science.1165115 18845749

