# Supplementary material for: Patterns of Species Richness and Turnover for the South American Rodent Fauna
Source: PLoS One. 2016 Mar 21;11(3):e0151895. doi: 10.1371/journal.pone.0151895 (PMC4801412; doi:10.1371/journal.pone.0151895)
Supplement: S1 Appendix — (DOCX) [file pone.0151895.s001.docx]

**Supporting Information**

**Appendix S1. Moran’s I correlogram for rodent richness and turnover.** Figures S1 to S6 depict the Moran’s correlogram for richness and turnover of all rodents, caviomorphs, and sigmodontiness. Moran’s correlograms were constructed based on 24 distance classes set up according to a criterion of equal numbers of pairs within each class. Significant values were identified through 250 permutations. All distance classes in all rodents, caviomorphs, and sigmodontines, for alpha and beta diversity, returned a significant p-value at α<0.05.


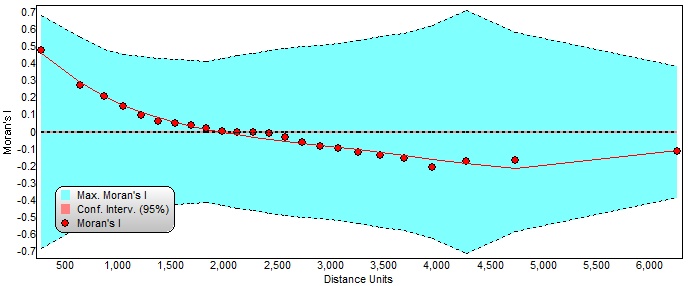


**Figure S1** Moran's I correlogram for richness of all rodents.


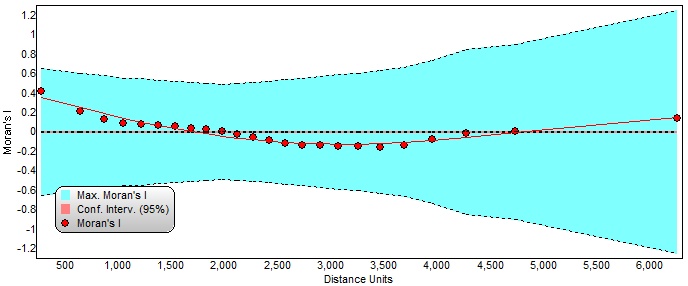


**Figure S2** Moran's I correlogram for turnover of all rodents.


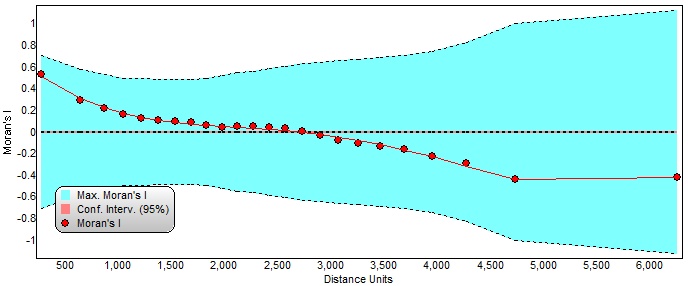


**Figure S3** Moran's I correlogram for richness of caviomorphs.


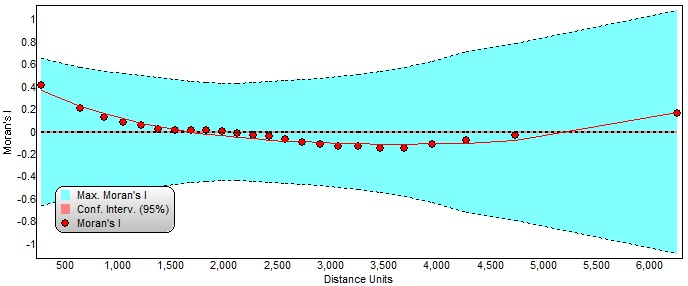


**Figure S4** Moran's I correlogram for turnover of caviomorphs.


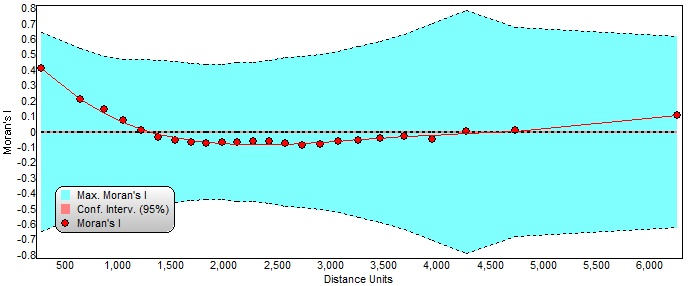


**Figure S5** Moran's I correlogram for richness of sigmodontines.


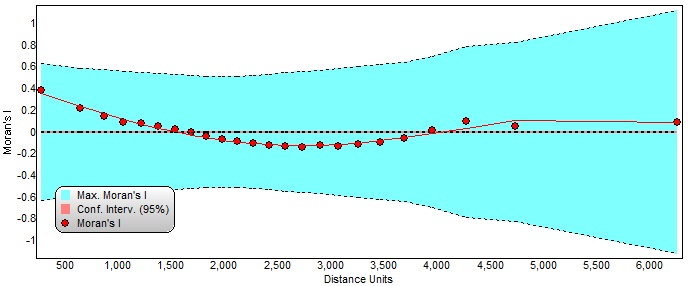


**Figure S6** Moran's I correlogram for turnover of sigmodontines.
