# Supplementary material for: Patterns of Species Richness and Turnover for the South American Rodent Fauna
Source: PLoS One. 2016 Mar 21;11(3):e0151895. doi: 10.1371/journal.pone.0151895 (PMC4801412; doi:10.1371/journal.pone.0151895)
Supplement: S2 Appendix — (DOCX) [file pone.0151895.s002.docx]

**Supporting Information**

**Appendix S2. Range sizes of rodent species.** The box below presented each South American rodent species, their membership in higher taxa, range sizes in km², and the information if the species is a restricted-range species (RR – with range size < 43,000 km², or the quartile of species with the smallest ranges).

| **Species** | **Range Size km²** | **Family** | **Tribe (Sigmodontines)** | **Superfamily (Caviomorphs)** | **RR** |
| --- | --- | --- | --- | --- | --- |
| *O_dariensis* | 24,227 | Geomyidae |  |  | Yes |
| *H_anomalus* | 484,874 | Heteromyidae |  |  |  |
| *H_australis* | 280,750 | Heteromyidae |  |  |  |
| *H_catopterius* | 39,594 | Heteromyidae |  |  | Yes |
| *H_desmarestianus* | 6,035 | Heteromyidae |  |  | Yes |
| *H_oasicus* | 3,012 | Heteromyidae |  |  | Yes |
| *H_teleus* | 35,960 | Heteromyidae |  |  | Yes |
| *G_aestuans* | 3,415,431 | Sciuridae |  |  |  |
| *G_brasiliensis* | 2,653,710 | Sciuridae |  |  |  |
| *H_igniventris* | 2,251,883 | Sciuridae |  |  |  |
| *H_pyrrhinus* | 1,261,138 | Sciuridae |  |  |  |
| *H_spadiceus* | 3,289,344 | Sciuridae |  |  |  |
| *M_flaviventer* | 2,672,822 | Sciuridae |  |  |  |
| *M_isthmius* | 89,010 | Sciuridae |  |  |  |
| *M_mimulus* | 14,790 | Sciuridae |  |  | Yes |
| *M_otinus* | 39,495 | Sciuridae |  |  | Yes |
| *M_sabanillae* | 27,605 | Sciuridae |  |  | Yes |
| *M_santanderensis* | 8,696 | Sciuridae |  |  | Yes |
| *M_similis* | 132,545 | Sciuridae |  |  |  |
| *M_simonsi* | 16,389 | Sciuridae |  |  | Yes |
| *N_granatensis* | 1,057,918 | Sciuridae |  |  |  |
| *N_pucheranii* | 2,847,307 | Sciuridae |  |  |  |
| *S_nebouxii* | 61,293 | Sciuridae |  |  |  |
| *S_pusillus* | 1,947,013 | Sciuridae |  |  |  |
| *S_stramineus* | 41,586 | Sciuridae |  |  | Yes |
| *Syntheosciurus_sp* | 21,519 | Sciuridae |  |  | Yes |
| *A_andina* | 546,156 | Cricetidae | Abrotrichini |  |  |
| *A_hershkovitzi* | 25,574 | Cricetidae | Abrotrichini |  | Yes |
| *A_illutea* | 59,774 | Cricetidae | Abrotrichini |  |  |
| *A_jelskii* | 710,632 | Cricetidae | Abrotrichini |  |  |
| *A_lanosa* | 283,682 | Cricetidae | Abrotrichini |  |  |
| *A_longipilis* | 1,155,414 | Cricetidae | Abrotrichini |  |  |
| *A_olivacea* | 1,585,401 | Cricetidae | Abrotrichini |  |  |
| *A_sanborni* | 90,351 | Cricetidae | Abrotrichini |  |  |
| *C_macronyx* | 550,231 | Cricetidae | Abrotrichini |  |  |
| *C_megalonyx* | 71,017 | Cricetidae | Abrotrichini |  |  |
| *G_valdivianus* | 610,488 | Cricetidae | Abrotrichini |  |  |
| *N_edwardsii* | 440,649 | Cricetidae | Abrotrichini |  |  |
| *P_annectens* | 19,341 | Cricetidae | Abrotrichini |  | Yes |
| *A_aerosus* | 252,666 | Cricetidae | Akodontini |  |  |
| *A_affinis* | 59,706 | Cricetidae | Akodontini |  |  |
| *A_albiventer* | 299,953 | Cricetidae | Akodontini |  |  |
| *A_azarae* | 1,455,726 | Cricetidae | Akodontini |  |  |
| *A_boliviensis* | 367,234 | Cricetidae | Akodontini |  |  |
| *A_budini* | 69,053 | Cricetidae | Akodontini |  |  |
| *A_caenosus* | 117,168 | Cricetidae | Akodontini |  |  |
| *A_cursor* | 1,017,938 | Cricetidae | Akodontini |  |  |
| *A_dayi* | 246,218 | Cricetidae | Akodontini |  |  |
| *A_dolores* | 987,217 | Cricetidae | Akodontini |  |  |
| *A_fumeus* | 260,109 | Cricetidae | Akodontini |  |  |
| *A_iniscatus* | 544,187 | Cricetidae | Akodontini |  |  |
| *A_josemariarguedasi* | 27,469 | Cricetidae | Akodontini |  | Yes |
| *A_juninensis* | 115,166 | Cricetidae | Akodontini |  |  |
| *A_kofordi* | 72,559 | Cricetidae | Akodontini |  |  |
| *A_lindberghi* | 242,971 | Cricetidae | Akodontini |  |  |
| *A_lutescens* | 225,739 | Cricetidae | Akodontini |  |  |
| *A_mimus* | 101,659 | Cricetidae | Akodontini |  |  |
| *A_mollis* | 209,292 | Cricetidae | Akodontini |  |  |
| *A_montensis* | 1,317,647 | Cricetidae | Akodontini |  |  |
| *A_mystax* | 21,775 | Cricetidae | Akodontini |  | Yes |
| *A_orophilus* | 36,233 | Cricetidae | Akodontini |  | Yes |
| *A_paranaensis* | 487,883 | Cricetidae | Akodontini |  |  |
| *A_pervalens* | 63,447 | Cricetidae | Akodontini |  |  |
| *A_philipmyersi* | 9,760 | Cricetidae | Akodontini |  | Yes |
| *A_polopi* | 46,307 | Cricetidae | Akodontini |  |  |
| *A_reigi* | 70,843 | Cricetidae | Akodontini |  |  |
| *A_sanctipaulensis* | 24,150 | Cricetidae | Akodontini |  | Yes |
| *A_serrensis* | 461,471 | Cricetidae | Akodontini |  |  |
| *A_siberiae* | 34,255 | Cricetidae | Akodontini |  | Yes |
| *A_simulator* | 195,697 | Cricetidae | Akodontini |  |  |
| *A_spegazzinii* | 217,144 | Cricetidae | Akodontini |  |  |
| *A_subfuscus* | 314,453 | Cricetidae | Akodontini |  |  |
| *A_surdus* | 16,218 | Cricetidae | Akodontini |  | Yes |
| *A_sylvanus* | 27,089 | Cricetidae | Akodontini |  | Yes |
| *A_toba* | 733,122 | Cricetidae | Akodontini |  |  |
| *A_torques* | 58,825 | Cricetidae | Akodontini |  |  |
| *A_varius* | 285,467 | Cricetidae | Akodontini |  |  |
| *Akodon_sp* | 337,146 | Cricetidae | Akodontini |  |  |
| *B_breviceps* | 636,802 | Cricetidae | Akodontini |  |  |
| *B_chacoensis* | 122,939 | Cricetidae | Akodontini |  |  |
| *B_griserufescens* | 52,147 | Cricetidae | Akodontini |  |  |
| *B_guarani* | 21,408 | Cricetidae | Akodontini |  | Yes |
| *B_igniventris* | 15,756 | Cricetidae | Akodontini |  | Yes |
| *B_iheringi* | 188,627 | Cricetidae | Akodontini |  |  |
| *B_labiosus* | 362,223 | Cricetidae | Akodontini |  |  |
| *B_misionensis* | 18,283 | Cricetidae | Akodontini |  | Yes |
| *B_paradisus* | 18,788 | Cricetidae | Akodontini |  | Yes |
| *B_soricinus* | 120,213 | Cricetidae | Akodontini |  |  |
| *B_torresi* | 18,761 | Cricetidae | Akodontini |  | Yes |
| *D_kempi* | 337,917 | Cricetidae | Akodontini |  |  |
| *G_fronto* | 71,662 | Cricetidae | Akodontini |  |  |
| *G_planaltensis* | 198,067 | Cricetidae | Akodontini |  |  |
| *J_candango* | 56,677 | Cricetidae | Akodontini |  |  |
| *J_huanchacae* | 128,250 | Cricetidae | Akodontini |  |  |
| *K_tomentosus* | 1,429,280 | Cricetidae | Akodontini |  |  |
| *L_apicalis* | 94,088 | Cricetidae | Akodontini |  |  |
| *N_amoenus* | 311,798 | Cricetidae | Akodontini |  |  |
| *N_lactens* | 295,757 | Cricetidae | Akodontini |  |  |
| *N_lasiurus* | 7,473,449 | Cricetidae | Akodontini |  |  |
| *N_lenguarum* | 859,364 | Cricetidae | Akodontini |  |  |
| *N_obscurus* | 50,227 | Cricetidae | Akodontini |  |  |
| *N_punctulatus* | 164,355 | Cricetidae | Akodontini |  |  |
| *N_urichi* | 1,426,790 | Cricetidae | Akodontini |  |  |
| *O_amazonicus* | 1,229,399 | Cricetidae | Akodontini |  |  |
| *O_caparaoe* | 20,658 | Cricetidae | Akodontini |  | Yes |
| *O_dasytrichus* | 1,170,852 | Cricetidae | Akodontini |  |  |
| *O_delator* | 3,167,534 | Cricetidae | Akodontini |  |  |
| *O_hiska* | 157,107 | Cricetidae | Akodontini |  |  |
| *O_hucucha* | 11,650 | Cricetidae | Akodontini |  | Yes |
| *O_inca* | 616,070 | Cricetidae | Akodontini |  |  |
| *O_josei* | 25,096 | Cricetidae | Akodontini |  | Yes |
| *O_juliacae* | 145,064 | Cricetidae | Akodontini |  |  |
| *O_nasutus* | 332,189 | Cricetidae | Akodontini |  |  |
| *O_nigrifrons* | 82,764 | Cricetidae | Akodontini |  |  |
| *O_paramensis* | 289,547 | Cricetidae | Akodontini |  |  |
| *O_quaestor* | 570,542 | Cricetidae | Akodontini |  |  |
| *O_rufus* | 841,914 | Cricetidae | Akodontini |  |  |
| *O_wayku* | 33,086 | Cricetidae | Akodontini |  | Yes |
| *S_aquaticus* | 347,515 | Cricetidae | Akodontini |  |  |
| *S_tumidus* | 491,204 | Cricetidae | Akodontini |  |  |
| *T_cerradensis* | 851,228 | Cricetidae | Akodontini |  |  |
| *T_lasiotis* | 1,470,485 | Cricetidae | Akodontini |  |  |
| *T_nigrita* | 946,574 | Cricetidae | Akodontini |  |  |
| *A_leander* | 10,986 | Cricetidae | Ichthyomyini |  | Yes |
| *C_orcesi* | 149,478 | Cricetidae | Ichthyomyini |  |  |
| *C_trichotis* | 70,917 | Cricetidae | Ichthyomyini |  |  |
| *I_hydrobates* | 166,715 | Cricetidae | Ichthyomyini |  |  |
| *I_pittieri* | 33,332 | Cricetidae | Ichthyomyini |  | Yes |
| *I_stolzmanni* | 237,736 | Cricetidae | Ichthyomyini |  |  |
| *I_tweedii* | 66,921 | Cricetidae | Ichthyomyini |  |  |
| *N_ferreirai* | 214,284 | Cricetidae | Ichthyomyini |  |  |
| *N_monticolus* | 255,805 | Cricetidae | Ichthyomyini |  |  |
| *N_mussoi* | 22,636 | Cricetidae | Ichthyomyini |  | Yes |
| *N_oyapocki* | 434,076 | Cricetidae | Ichthyomyini |  |  |
| *N_peruviensis* | 50,700 | Cricetidae | Ichthyomyini |  |  |
| *N_venezuelae* | 649,557 | Cricetidae | Ichthyomyini |  |  |
| *P_roraimae* | 18,824 | Cricetidae | Ichthyomyini |  | Yes |
| *A_chebezi* | 56,053 | Cricetidae | incertae sedis |  |  |
| *A_edax* | 579,279 | Cricetidae | incertae sedis |  |  |
| *A_ruschii* | 360,985 | Cricetidae | incertae sedis |  |  |
| *C_sahamae* | 265,358 | Cricetidae | incertae sedis |  |  |
| *D_dorsalis* | 514,333 | Cricetidae | incertae sedis |  |  |
| *D_sublineatus* | 457,613 | Cricetidae | incertae sedis |  |  |
| *E_chinchilloides* | 64,288 | Cricetidae | incertae sedis |  |  |
| *E_fossor* | 30,026 | Cricetidae | incertae sedis |  | Yes |
| *E_mordax* | 85,185 | Cricetidae | incertae sedis |  |  |
| *E_petersoni* | 647,412 | Cricetidae | incertae sedis |  |  |
| *I_tarsalis* | 238,561 | Cricetidae | incertae sedis |  |  |
| *J_ossitenuis* | 215,996 | Cricetidae | incertae sedis |  |  |
| *J_pictipes* | 669,028 | Cricetidae | incertae sedis |  |  |
| *J_rimofrons* | 13,174 | Cricetidae | incertae sedis |  | Yes |
| *N_bogotensis* | 57,124 | Cricetidae | incertae sedis |  |  |
| *N_ebriosus* | 983,884 | Cricetidae | incertae sedis |  |  |
| *N_latebricola* | 32,494 | Cricetidae | incertae sedis |  | Yes |
| *P_ferrugineus* | 41,024 | Cricetidae | incertae sedis |  | Yes |
| *P_kofordi* | 45,457 | Cricetidae | incertae sedis |  |  |
| *P_lemminus* | 55,851 | Cricetidae | incertae sedis |  |  |
| *W_oenax* | 506,172 | Cricetidae | incertae sedis |  |  |
| *A_galapagoensis* | 303 | Cricetidae | Oryzomyini |  | Yes |
| *A_savamis* | 22,262 | Cricetidae | Oryzomyini |  | Yes |
| *A_xanthaeolus* | 441,557 | Cricetidae | Oryzomyini |  |  |
| *C_goytaca* | 10,168 | Cricetidae | Oryzomyini |  | Yes |
| *C_langguthi* | 566,796 | Cricetidae | Oryzomyini |  |  |
| *C_maracajuensis* | 2,261,084 | Cricetidae | Oryzomyini |  |  |
| *C_marinhus* | 55,829 | Cricetidae | Oryzomyini |  |  |
| *C_scotti* | 2,543,721 | Cricetidae | Oryzomyini |  |  |
| *C_subflavus* | 819,260 | Cricetidae | Oryzomyini |  |  |
| *C_vivoi* | 588,227 | Cricetidae | Oryzomyini |  |  |
| *D_albimaculatus* | 141,739 | Cricetidae | Oryzomyini |  |  |
| *E_emmonsae* | 610,821 | Cricetidae | Oryzomyini |  |  |
| *E_lamia* | 193,021 | Cricetidae | Oryzomyini |  |  |
| *E_legatus* | 251,517 | Cricetidae | Oryzomyini |  |  |
| *E_macconnelli* | 5,879,653 | Cricetidae | Oryzomyini |  |  |
| *E_nitidus* | 1,820,334 | Cricetidae | Oryzomyini |  |  |
| *E_polius* | 48,061 | Cricetidae | Oryzomyini |  |  |
| *E_russatus* | 1,855,704 | Cricetidae | Oryzomyini |  |  |
| *H_acritus* | 220,766 | Cricetidae | Oryzomyini |  |  |
| *H_alfaroi* | 415,549 | Cricetidae | Oryzomyini |  |  |
| *H_brasiliensis* | 560,532 | Cricetidae | Oryzomyini |  |  |
| *H_chacarius* | 1,332,585 | Cricetidae | Oryzomyini |  |  |
| *H_fuscatus* | 18,651 | Cricetidae | Oryzomyini |  | Yes |
| *H_intectus* | 13,132 | Cricetidae | Oryzomyini |  | Yes |
| *H_lagigliai* | 15,302 | Cricetidae | Oryzomyini |  | Yes |
| *H_laticeps* | 273,594 | Cricetidae | Oryzomyini |  |  |
| *H_megacephalus* | 6,485,969 | Cricetidae | Oryzomyini |  |  |
| *H_oniscus* | 76,953 | Cricetidae | Oryzomyini |  |  |
| *H_perenensis* | 2,187,051 | Cricetidae | Oryzomyini |  |  |
| *H_sciureus* | 7,906,350 | Cricetidae | Oryzomyini |  |  |
| *H_tatei* | 17,620 | Cricetidae | Oryzomyini |  | Yes |
| *H_venezuelae* | 433,872 | Cricetidae | Oryzomyini |  |  |
| *H_vulpinus* | 1,036,054 | Cricetidae | Oryzomyini |  |  |
| *H_yunganus* | 6,281,253 | Cricetidae | Oryzomyini |  |  |
| *L_molitor* | 202,576 | Cricetidae | Oryzomyini |  |  |
| *M_altissimus* | 391,720 | Cricetidae | Oryzomyini |  |  |
| *M_caliginosus* | 413,157 | Cricetidae | Oryzomyini |  |  |
| *M_columbianus* | 39,177 | Cricetidae | Oryzomyini |  | Yes |
| *M_hammondi* | 17,154 | Cricetidae | Oryzomyini |  | Yes |
| *M_minutus* | 812,097 | Cricetidae | Oryzomyini |  |  |
| *M_robustulus* | 124,762 | Cricetidae | Oryzomyini |  |  |
| *M_transitorius* | 21,463 | Cricetidae | Oryzomyini |  | Yes |
| *M_zunigae* | 11,149 | Cricetidae | Oryzomyini |  | Yes |
| *N_albigularis* | 142,758 | Cricetidae | Oryzomyini |  |  |
| *N_apicalis* | 940,123 | Cricetidae | Oryzomyini |  |  |
| *N_auriventer* | 129,186 | Cricetidae | Oryzomyini |  |  |
| *N_caracolus* | 27,403 | Cricetidae | Oryzomyini |  | Yes |
| *N_childi* | 141,932 | Cricetidae | Oryzomyini |  |  |
| *N_darwini* | 900 | Cricetidae | Oryzomyini |  | Yes |
| *N_dubosti* | 268,174 | Cricetidae | Oryzomyini |  |  |
| *N_fernandinae* | 418 | Cricetidae | Oryzomyini |  | Yes |
| *N_g_grandis* | 32,603 | Cricetidae | Oryzomyini |  | Yes |
| *N_guianae* | 477,945 | Cricetidae | Oryzomyini |  |  |
| *N_indefessus* | 848 | Cricetidae | Oryzomyini |  | Yes |
| *N_keaysi* | 306,151 | Cricetidae | Oryzomyini |  |  |
| *N_levipes* | 141,280 | Cricetidae | Oryzomyini |  |  |
| *N_maculiventer* | 20,229 | Cricetidae | Oryzomyini |  | Yes |
| *N_meridensis* | 65,576 | Cricetidae | Oryzomyini |  |  |
| *N_minutus* | 335,337 | Cricetidae | Oryzomyini |  |  |
| *N_moerex* | 8,364 | Cricetidae | Oryzomyini |  | Yes |
| *N_musseri* | 221,072 | Cricetidae | Oryzomyini |  |  |
| *N_narboroughi* | 341 | Cricetidae | Oryzomyini |  | Yes |
| *N_nimbosus* | 18,332 | Cricetidae | Oryzomyini |  | Yes |
| *N_p_palmipes* | 5,822 | Cricetidae | Oryzomyini |  | Yes |
| *N_p_tatei* | 33,656 | Cricetidae | Oryzomyini |  | Yes |
| *N_paracou* | 1,065,939 | Cricetidae | Oryzomyini |  |  |
| *N_pectoralis* | 118,847 | Cricetidae | Oryzomyini |  |  |
| *N_rattus* | 9,687,543 | Cricetidae | Oryzomyini |  |  |
| *N_spinosus* | 3,997,135 | Cricetidae | Oryzomyini |  |  |
| *N_squamipes* | 1,927,498 | Cricetidae | Oryzomyini |  |  |
| *N_swarthi* | 594 | Cricetidae | Oryzomyini |  | Yes |
| *N_tenuipes* | 293,127 | Cricetidae | Oryzomyini |  |  |
| *Nectomys_grandis* | 113,500 | Cricetidae | Oryzomyini |  |  |
| *O_andinus* | 229,760 | Cricetidae | Oryzomyini |  |  |
| *O_arenalis* | 216,855 | Cricetidae | Oryzomyini |  |  |
| *O_auyantepui* | 1,310,293 | Cricetidae | Oryzomyini |  |  |
| *O_balneator* | 70,241 | Cricetidae | Oryzomyini |  |  |
| *O_bicolor* | 8,258,727 | Cricetidae | Oryzomyini |  |  |
| *O_brendae* | 112,167 | Cricetidae | Oryzomyini |  |  |
| *O_catherinae* | 1,470,321 | Cricetidae | Oryzomyini |  |  |
| *O_chacoensis* | 850,398 | Cricetidae | Oryzomyini |  |  |
| *O_cleberi* | 636,692 | Cricetidae | Oryzomyini |  |  |
| *O_concolor* | 948,944 | Cricetidae | Oryzomyini |  |  |
| *O_couesi* | 58,415 | Cricetidae | Oryzomyini |  |  |
| *O_delicatus* | 886,330 | Cricetidae | Oryzomyini |  |  |
| *O_destructor* | 908,962 | Cricetidae | Oryzomyini |  |  |
| *O_flavescens* | 2,998,800 | Cricetidae | Oryzomyini |  |  |
| *O_flavicans* | 440,015 | Cricetidae | Oryzomyini |  |  |
| *O_fornesi* | 22,280 | Cricetidae | Oryzomyini |  | Yes |
| *O_gorgasi* | 171,005 | Cricetidae | Oryzomyini |  |  |
| *O_griseolus* | 14,183 | Cricetidae | Oryzomyini |  | Yes |
| *O_longicaudatus* | 957,670 | Cricetidae | Oryzomyini |  |  |
| *O_magellanicus* | 215,404 | Cricetidae | Oryzomyini |  |  |
| *O_mamorae* | 1,455,448 | Cricetidae | Oryzomyini |  |  |
| *O_mattogrossae* | 1,436,684 | Cricetidae | Oryzomyini |  |  |
| *O_messorius* | 550,657 | Cricetidae | Oryzomyini |  |  |
| *O_microtis* | 2,457,941 | Cricetidae | Oryzomyini |  |  |
| *O_moojeni* | 221,557 | Cricetidae | Oryzomyini |  |  |
| *O_nigripes* | 2,459,421 | Cricetidae | Oryzomyini |  |  |
| *O_paricola* | 3,193,732 | Cricetidae | Oryzomyini |  |  |
| *O_phaeotis* | 483,063 | Cricetidae | Oryzomyini |  |  |
| *O_rex* | 1,309,244 | Cricetidae | Oryzomyini |  |  |
| *O_roberti* | 6,094,553 | Cricetidae | Oryzomyini |  |  |
| *O_rupestris* | 497,448 | Cricetidae | Oryzomyini |  |  |
| *O_rutilus* | 1,018,612 | Cricetidae | Oryzomyini |  |  |
| *O_speciosus* | 591,171 | Cricetidae | Oryzomyini |  |  |
| *O_stramineus* | 1,112,290 | Cricetidae | Oryzomyini |  |  |
| *O_superans* | 1,298,882 | Cricetidae | Oryzomyini |  |  |
| *O_sydandersoni* | 120,337 | Cricetidae | Oryzomyini |  |  |
| *O_trinitatis* | 8,496,080 | Cricetidae | Oryzomyini |  |  |
| *O_utiaritensis* | 546,068 | Cricetidae | Oryzomyini |  |  |
| *P_simplex* | 3,776,962 | Cricetidae | Oryzomyini |  |  |
| *S_alfari* | 404,011 | Cricetidae | Oryzomyini |  |  |
| *S_angouya* | 1,467,951 | Cricetidae | Oryzomyini |  |  |
| *S_melanops* | 157,785 | Cricetidae | Oryzomyini |  |  |
| *S_ucayalensis* | 536,353 | Cricetidae | Oryzomyini |  |  |
| *T_aphrastus* | 11,482 | Cricetidae | Oryzomyini |  | Yes |
| *T_bolivaris* | 201,236 | Cricetidae | Oryzomyini |  |  |
| *T_talamancae* | 713,870 | Cricetidae | Oryzomyini |  |  |
| *Z_brevicauda* | 2,673,751 | Cricetidae | Oryzomyini |  |  |
| *Z_brunneus* | 104,738 | Cricetidae | Oryzomyini |  |  |
| *A_olrogi* | 22,617 | Cricetidae | Phyllotini |  | Yes |
| *A_pearsoni* | 136,784 | Cricetidae | Phyllotini |  |  |
| *A_pictus* | 435,601 | Cricetidae | Phyllotini |  |  |
| *A_roigi* | 115,168 | Cricetidae | Phyllotini |  |  |
| *A_sublimis* | 446,243 | Cricetidae | Phyllotini |  |  |
| *Auliscomys_boliviensis* | 125,397 | Cricetidae | Phyllotini |  |  |
| *C_apicalis* | 13,120 | Cricetidae | Phyllotini |  | Yes |
| *C_callidus* | 819,241 | Cricetidae | Phyllotini |  |  |
| *C_callosus* | 1,454,043 | Cricetidae | Phyllotini |  |  |
| *C_cerqueirai* | 48,364 | Cricetidae | Phyllotini |  |  |
| *C_expulsus* | 1,238,577 | Cricetidae | Phyllotini |  |  |
| *C_hummelincki* | 388,603 | Cricetidae | Phyllotini |  |  |
| *C_laucha* | 2,490,235 | Cricetidae | Phyllotini |  |  |
| *C_lepidus* | 881,915 | Cricetidae | Phyllotini |  |  |
| *C_musculinus* | 2,716,001 | Cricetidae | Phyllotini |  |  |
| *C_sorellus* | 292,036 | Cricetidae | Phyllotini |  |  |
| *C_tener* | 2,718,279 | Cricetidae | Phyllotini |  |  |
| *C_tocantinsi* | 223,044 | Cricetidae | Phyllotini |  |  |
| *C_venustus* | 175,719 | Cricetidae | Phyllotini |  |  |
| *Calomys_boliviae* | 266,457 | Cricetidae | Phyllotini |  |  |
| *E_bolsonensis* | 84,304 | Cricetidae | Phyllotini |  |  |
| *E_dunaris* | 23,733 | Cricetidae | Phyllotini |  | Yes |
| *E_hirtipes* | 240,447 | Cricetidae | Phyllotini |  |  |
| *E_moreni* | 276,958 | Cricetidae | Phyllotini |  |  |
| *E_morgani* | 661,763 | Cricetidae | Phyllotini |  |  |
| *E_puerulus* | 206,441 | Cricetidae | Phyllotini |  |  |
| *E_typus* | 989,215 | Cricetidae | Phyllotini |  |  |
| *G_chacoensis* | 1,220,355 | Cricetidae | Phyllotini |  |  |
| *G_domorum* | 253,794 | Cricetidae | Phyllotini |  |  |
| *G_edithae* | 29,457 | Cricetidae | Phyllotini |  | Yes |
| *G_garleppii* | 70,532 | Cricetidae | Phyllotini |  |  |
| *G_griseoflavus* | 1,387,979 | Cricetidae | Phyllotini |  |  |
| *L_micropus* | 666,425 | Cricetidae | Phyllotini |  |  |
| *P_alisosiensis* | 29,624 | Cricetidae | Phyllotini |  | Yes |
| *P_amicus* | 230,122 | Cricetidae | Phyllotini |  |  |
| *P_andium* | 285,497 | Cricetidae | Phyllotini |  |  |
| *P_anitae* | 31,167 | Cricetidae | Phyllotini |  | Yes |
| *P_bonariensis* | 30,508 | Cricetidae | Phyllotini |  | Yes |
| *P_caprinus* | 84,083 | Cricetidae | Phyllotini |  |  |
| *P_darwini* | 223,656 | Cricetidae | Phyllotini |  |  |
| *P_definitus* | 18,309 | Cricetidae | Phyllotini |  | Yes |
| *P_gerbillus* | 37,236 | Cricetidae | Phyllotini |  | Yes |
| *P_haggardi* | 23,814 | Cricetidae | Phyllotini |  | Yes |
| *P_limatus* | 305,237 | Cricetidae | Phyllotini |  |  |
| *P_magister* | 277,454 | Cricetidae | Phyllotini |  |  |
| *P_osgoodi* | 17,386 | Cricetidae | Phyllotini |  | Yes |
| *P_osilae* | 557,273 | Cricetidae | Phyllotini |  |  |
| *P_xanthopygus* | 2,250,743 | Cricetidae | Phyllotini |  |  |
| *S_delicatus* | 170,812 | Cricetidae | Phyllotini |  |  |
| *T_primus* | 40,013 | Cricetidae | Phyllotini |  | Yes |
| *T_wolffsohni* | 152,520 | Cricetidae | Phyllotini |  |  |
| *R_auritus* | 1,680,505 | Cricetidae | Reithrodontini |  |  |
| *R_typicus* | 338,660 | Cricetidae | Reithrodontini |  |  |
| *S_alstoni* | 1,584,424 | Cricetidae | Sigmodontini |  |  |
| *S_hirsutus* | 542,943 | Cricetidae | Sigmodontini |  |  |
| *S_inopinatus* | 16,302 | Cricetidae | Sigmodontini |  | Yes |
| *S_peruanus* | 77,340 | Cricetidae | Sigmodontini |  |  |
| *A_lugens* | 26,725 | Cricetidae | Thomasomyini |  | Yes |
| *Aepeomys_reigi* | 11,713 | Cricetidae | Thomasomyini |  | Yes |
| *C_fumeus* | 46,035 | Cricetidae | Thomasomyini |  |  |
| *C_instans* | 268,032 | Cricetidae | Thomasomyini |  |  |
| *R_austrinus* | 173,319 | Cricetidae | Thomasomyini |  |  |
| *R_cariri* | 261,111 | Cricetidae | Thomasomyini |  |  |
| *R_caucensis* | 69,252 | Cricetidae | Thomasomyini |  |  |
| *R_couesi* | 223,621 | Cricetidae | Thomasomyini |  |  |
| *R_emiliae* | 1,268,242 | Cricetidae | Thomasomyini |  |  |
| *R_fulviventer* | 62,875 | Cricetidae | Thomasomyini |  |  |
| *R_gardneri* | 383,799 | Cricetidae | Thomasomyini |  |  |
| *R_ipukensis* | 87,259 | Cricetidae | Thomasomyini |  |  |
| *R_itoan* | 109,216 | Cricetidae | Thomasomyini |  |  |
| *R_latimanus* | 333,877 | Cricetidae | Thomasomyini |  |  |
| *R_leucodactylus* | 5,214,252 | Cricetidae | Thomasomyini |  |  |
| *R_longilingua* | 94,461 | Cricetidae | Thomasomyini |  |  |
| *R_macconnelli* | 134,297 | Cricetidae | Thomasomyini |  |  |
| *R_macrurus* | 1,898,998 | Cricetidae | Thomasomyini |  |  |
| *R_mastacalis* | 752,539 | Cricetidae | Thomasomyini |  |  |
| *R_modicus* | 72,977 | Cricetidae | Thomasomyini |  |  |
| *R_nitela* | 1,591,548 | Cricetidae | Thomasomyini |  |  |
| *R_ochrogaster* | 16,807 | Cricetidae | Thomasomyini |  | Yes |
| *R_rufescens* | 379,040 | Cricetidae | Thomasomyini |  |  |
| *R_similis* | 49,192 | Cricetidae | Thomasomyini |  |  |
| *R_tenuicauda* | 11,577 | Cricetidae | Thomasomyini |  | Yes |
| *R_tribei* | 39,404 | Cricetidae | Thomasomyini |  | Yes |
| *R_venezuelae* | 216,021 | Cricetidae | Thomasomyini |  |  |
| *R_venustus* | 47,378 | Cricetidae | Thomasomyini |  |  |
| *R_wetzeli* | 130,324 | Cricetidae | Thomasomyini |  |  |
| *Rhagomys_sp* | 49,112 | Cricetidae | Thomasomyini |  |  |
| *T_andersoni* | 41,237 | Cricetidae | Thomasomyini |  | Yes |
| *T_apeco* | 21,903 | Cricetidae | Thomasomyini |  | Yes |
| *T_aureus* | 628,600 | Cricetidae | Thomasomyini |  |  |
| *T_auricularis* | 35,203 | Cricetidae | Thomasomyini |  | Yes |
| *T_australis* | 29,286 | Cricetidae | Thomasomyini |  | Yes |
| *T_baeops* | 232,923 | Cricetidae | Thomasomyini |  |  |
| *T_bombycinus* | 20,922 | Cricetidae | Thomasomyini |  | Yes |
| *T_caudivarius* | 65,610 | Cricetidae | Thomasomyini |  |  |
| *T_cinereiventer* | 43,633 | Cricetidae | Thomasomyini |  |  |
| *T_cinereus* | 74,436 | Cricetidae | Thomasomyini |  |  |
| *T_cinnameus* | 168,516 | Cricetidae | Thomasomyini |  |  |
| *T_contradictus* | 50,889 | Cricetidae | Thomasomyini |  |  |
| *T_daphne* | 136,917 | Cricetidae | Thomasomyini |  |  |
| *T_dispar* | 26,512 | Cricetidae | Thomasomyini |  | Yes |
| *T_eleusis* | 31,737 | Cricetidae | Thomasomyini |  | Yes |
| *T_emeritus* | 25,102 | Cricetidae | Thomasomyini |  | Yes |
| *T_erro* | 18,117 | Cricetidae | Thomasomyini |  | Yes |
| *T_fumeus* | 9,307 | Cricetidae | Thomasomyini |  | Yes |
| *T_gracilis* | 44,887 | Cricetidae | Thomasomyini |  |  |
| *T_hudsoni* | 14,350 | Cricetidae | Thomasomyini |  | Yes |
| *T_hylophilus* | 22,364 | Cricetidae | Thomasomyini |  | Yes |
| *T_incanus* | 92,433 | Cricetidae | Thomasomyini |  |  |
| *T_ischyrus* | 66,933 | Cricetidae | Thomasomyini |  |  |
| *T_kalinowskii* | 102,830 | Cricetidae | Thomasomyini |  |  |
| *T_ladewi* | 48,270 | Cricetidae | Thomasomyini |  |  |
| *T_laniger* | 53,627 | Cricetidae | Thomasomyini |  |  |
| *T_macrotis* | 16,077 | Cricetidae | Thomasomyini |  | Yes |
| *T_monochromos* | 7,446 | Cricetidae | Thomasomyini |  | Yes |
| *T_nicefori* | 46,805 | Cricetidae | Thomasomyini |  |  |
| *T_niveipes* | 28,032 | Cricetidae | Thomasomyini |  | Yes |
| *T_notatus* | 194,311 | Cricetidae | Thomasomyini |  |  |
| *T_onkiro* | 22,973 | Cricetidae | Thomasomyini |  | Yes |
| *T_oreas* | 214,083 | Cricetidae | Thomasomyini |  |  |
| *T_paramorum* | 69,352 | Cricetidae | Thomasomyini |  |  |
| *T_popayanus* | 35,233 | Cricetidae | Thomasomyini |  | Yes |
| *T_praetor* | 49,830 | Cricetidae | Thomasomyini |  |  |
| *T_princeps* | 16,138 | Cricetidae | Thomasomyini |  | Yes |
| *T_pyrrhonotus* | 52,191 | Cricetidae | Thomasomyini |  |  |
| *T_rosalinda* | 18,931 | Cricetidae | Thomasomyini |  | Yes |
| *T_silvestris* | 38,848 | Cricetidae | Thomasomyini |  | Yes |
| *T_taczanowskii* | 149,462 | Cricetidae | Thomasomyini |  |  |
| *T_ucucha* | 16,917 | Cricetidae | Thomasomyini |  | Yes |
| *T_vestitus* | 24,868 | Cricetidae | Thomasomyini |  | Yes |
| *T_vulcani* | 23,507 | Cricetidae | Thomasomyini |  | Yes |
| *W_cerradensis* | 220,465 | Cricetidae | Thomasomyini |  |  |
| *W_pyrrhorhinos* | 530,563 | Cricetidae | Thomasomyini |  |  |
| *C_aperea* | 6,575,296 | Caviidae |  | Cavioidea |  |
| *C_fulgida* | 763,762 | Caviidae |  | Cavioidea |  |
| *C_intermedia* | 6,594 | Caviidae |  | Cavioidea | Yes |
| *C_magna* | 110,013 | Caviidae |  | Cavioidea |  |
| *C_paca* | 13,472,855 | Cuniculidae |  | Cavioidea |  |
| *C_patzelti* | 17,268 | Caviidae |  | Cavioidea | Yes |
| *C_taczanowskii* | 836,245 | Cuniculidae |  | Cavioidea |  |
| *C_tschudii* | 924,033 | Caviidae |  | Cavioidea |  |
| *D_azarae* | 3,801,213 | Dasyproctidae |  | Cavioidea |  |
| *D_croconota* | 388,295 | Dasyproctidae |  | Cavioidea |  |
| *D_fuliginosa* | 3,409,373 | Dasyproctidae |  | Cavioidea |  |
| *D_guamara* | 8,803 | Dasyproctidae |  | Cavioidea | Yes |
| *D_iacki* | 40,813 | Dasyproctidae |  | Cavioidea | Yes |
| *D_kalinowskii* | 54,254 | Dasyproctidae |  | Cavioidea |  |
| *D_leporina* | 4,836,314 | Dasyproctidae |  | Cavioidea |  |
| *D_patagonum* | 1,300,159 | Caviidae |  | Cavioidea |  |
| *D_prymnolopha* | 2,001,873 | Dasyproctidae |  | Cavioidea |  |
| *D_punctata* | 744,559 | Dasyproctidae |  | Cavioidea |  |
| *D_salinicola* | 731,789 | Caviidae |  | Cavioidea |  |
| *D_variegata* | 438,515 | Dasyproctidae |  | Cavioidea |  |
| *G_comes* | 135,471 | Caviidae |  | Cavioidea |  |
| *G_flavidens* | 46,369 | Caviidae |  | Cavioidea |  |
| *G_leucoblephara* | 2,018,295 | Caviidae |  | Cavioidea |  |
| *G_musteloides* | 292,476 | Caviidae |  | Cavioidea |  |
| *G_spixii* | 1,707,066 | Caviidae |  | Cavioidea |  |
| *H_hydrochaeris* | 13,534,101 | Caviidae |  | Cavioidea |  |
| *H_isthmius* | 337,601 | Caviidae |  | Cavioidea |  |
| *K_acrobata* | 75,812 | Caviidae |  | Cavioidea |  |
| *K_rupestris* | 761,350 | Caviidae |  | Cavioidea |  |
| *M_acouchy* | 1,082,813 | Dasyproctidae |  | Cavioidea |  |
| *M_australis* | 1,911,418 | Caviidae |  | Cavioidea |  |
| *M_niata* | 168,048 | Caviidae |  | Cavioidea |  |
| *M_pratti* | 3,324,728 | Dasyproctidae |  | Cavioidea |  |
| *M_shiptoni* | 42,649 | Caviidae |  | Cavioidea | Yes |
| *C_chinchilla* | 489,377 | Chinchillidae |  | Chinchilloidea |  |
| *C_lanigera* | 33,782 | Chinchillidae |  | Chinchilloidea | Yes |
| *D_branickii* | 1,716,807 | Dinomyidae |  | Chinchilloidea |  |
| *L_ahuacaense* | 15,242 | Chinchillidae |  | Chinchilloidea | Yes |
| *L_crassus* | 47,279 | Chinchillidae |  | Chinchilloidea |  |
| *L_maximus* | 1,477,712 | Chinchillidae |  | Chinchilloidea |  |
| *L_viscacia* | 1,957,976 | Chinchillidae |  | Chinchilloidea |  |
| *L_wolffsohni* | 60,195 | Chinchillidae |  | Chinchilloidea |  |
| *C_bicolor* | 540,218 | Erethizontidae |  | Erethizontoidea |  |
| *C_ichillus* | 148,879 | Erethizontidae |  | Erethizontoidea |  |
| *C_insidiosus* | 168,410 | Erethizontidae |  | Erethizontoidea |  |
| *C_melanurus* | 1,465,296 | Erethizontidae |  | Erethizontoidea |  |
| *C_nycthemera* | 342,984 | Erethizontidae |  | Erethizontoidea |  |
| *C_prehensilis* | 13,044,939 | Erethizontidae |  | Erethizontoidea |  |
| *C_pruinosus* | 247,446 | Erethizontidae |  | Erethizontoidea |  |
| *C_quichua* | 507,609 | Erethizontidae |  | Erethizontoidea |  |
| *C_roosmalenorum* | 164,725 | Erethizontidae |  | Erethizontoidea |  |
| *C_rufescens* | 495,759 | Erethizontidae |  | Erethizontoidea |  |
| *C_speratus* | 19,788 | Erethizontidae |  | Erethizontoidea | Yes |
| *C_spinosus* | 1,193,740 | Erethizontidae |  | Erethizontoidea |  |
| *C_subspinosus* | 259,724 | Erethizontidae |  | Erethizontoidea |  |
| *C_vestitus* | 16,332 | Erethizontidae |  | Erethizontoidea | Yes |
| *A_bennettii* | 146,536 | Abrocomidae |  | Octodontoidea |  |
| *A_cinerea* | 510,834 | Abrocomidae |  | Octodontoidea |  |
| *A_famatina* | 7,084 | Abrocomidae |  | Octodontoidea | Yes |
| *A_fuscus* | 43,753 | Octodontidae |  | Octodontoidea |  |
| *A_porteri* | 23,434 | Octodontidae |  | Octodontoidea | Yes |
| *A_sagei* | 50,467 | Octodontidae |  | Octodontoidea |  |
| *A_schistacea* | 9,165 | Abrocomidae |  | Octodontoidea | Yes |
| *A_uspallata* | 5,226 | Abrocomidae |  | Octodontoidea | Yes |
| *A_vaccarum* | 5,511 | Abrocomidae |  | Octodontoidea | Yes |
| *Abrocoma_boliviensis* | 17,659 | Abrocomidae |  | Octodontoidea | Yes |
| *Abrocoma_budini* | 6,709 | Abrocomidae |  | Octodontoidea | Yes |
| *C_argentinus* | 245,465 | Ctenomyidae |  | Octodontoidea |  |
| *C_ashaninka* | 15,559 | Abrocomidae |  | Octodontoidea | Yes |
| *C_australis* | 43,373 | Ctenomyidae |  | Octodontoidea | Yes |
| *C_azarae* | 263,598 | Ctenomyidae |  | Octodontoidea |  |
| *C_bergi* | 84,794 | Ctenomyidae |  | Octodontoidea |  |
| *C_boliviensis* | 69,905 | Ctenomyidae |  | Octodontoidea |  |
| *C_bonettoi* | 36,890 | Ctenomyidae |  | Octodontoidea | Yes |
| *C_brasiliensis* | 8,276 | Ctenomyidae |  | Octodontoidea | Yes |
| *C_colburni* | 203,097 | Ctenomyidae |  | Octodontoidea |  |
| *C_coludo* | 46,286 | Ctenomyidae |  | Octodontoidea |  |
| *C_conoveri* | 118,328 | Ctenomyidae |  | Octodontoidea |  |
| *C_coyhaiquensis* | 16,876 | Ctenomyidae |  | Octodontoidea | Yes |
| *C_dorbignyi* | 90,295 | Ctenomyidae |  | Octodontoidea |  |
| *C_dorsalis* | 47,094 | Ctenomyidae |  | Octodontoidea |  |
| *C_emilianus* | 26,832 | Ctenomyidae |  | Octodontoidea | Yes |
| *C_famosus* | 45,080 | Ctenomyidae |  | Octodontoidea |  |
| *C_flamarioni* | 50,026 | Ctenomyidae |  | Octodontoidea |  |
| *C_fochi* | 40,544 | Ctenomyidae |  | Octodontoidea | Yes |
| *C_fodax* | 44,401 | Ctenomyidae |  | Octodontoidea |  |
| *C_frater* | 210,698 | Ctenomyidae |  | Octodontoidea |  |
| *C_fulvus* | 107,589 | Ctenomyidae |  | Octodontoidea |  |
| *C_goodfellowi* | 54,533 | Ctenomyidae |  | Octodontoidea |  |
| *C_haigi* | 231,022 | Ctenomyidae |  | Octodontoidea |  |
| *C_ibicuiensis* | 12,959 | Ctenomyidae |  | Octodontoidea | Yes |
| *C_johannis* | 46,599 | Ctenomyidae |  | Octodontoidea |  |
| *C_juris* | 47,798 | Ctenomyidae |  | Octodontoidea |  |
| *C_knighti* | 47,476 | Ctenomyidae |  | Octodontoidea |  |
| *C_lami* | 17,432 | Ctenomyidae |  | Octodontoidea | Yes |
| *C_laticeps* | 1,808,980 | Echimyidae |  | Octodontoidea |  |
| *C_latro* | 87,430 | Ctenomyidae |  | Octodontoidea |  |
| *C_leucodon* | 78,893 | Ctenomyidae |  | Octodontoidea |  |
| *C_lewisi* | 58,319 | Ctenomyidae |  | Octodontoidea |  |
| *C_magellanicus* | 274,820 | Ctenomyidae |  | Octodontoidea |  |
| *C_mariafarelli* | 62,096 | Ctenomyidae |  | Octodontoidea |  |
| *C_maulinus* | 92,568 | Ctenomyidae |  | Octodontoidea |  |
| *C_mendocinus* | 426,738 | Ctenomyidae |  | Octodontoidea |  |
| *C_minutus* | 56,522 | Ctenomyidae |  | Octodontoidea |  |
| *C_nattereri* | 245,722 | Ctenomyidae |  | Octodontoidea |  |
| *C_oblativus* | 19,250 | Abrocomidae |  | Octodontoidea | Yes |
| *C_occultus* | 46,479 | Ctenomyidae |  | Octodontoidea |  |
| *C_opimus* | 316,073 | Ctenomyidae |  | Octodontoidea |  |
| *C_osvaldoreigi* | 46,574 | Ctenomyidae |  | Octodontoidea |  |
| *C_paraguayensis* | 49,312 | Ctenomyidae |  | Octodontoidea |  |
| *C_pearsoni* | 40,046 | Ctenomyidae |  | Octodontoidea | Yes |
| *C_perrensi* | 1,853 | Ctenomyidae |  | Octodontoidea | Yes |
| *C_perrensi_complex* | 52,532 | Ctenomyidae |  | Octodontoidea |  |
| *C_peruanus* | 73,054 | Ctenomyidae |  | Octodontoidea |  |
| *C_pictus* | 37,312 | Echimyidae |  | Octodontoidea | Yes |
| *C_pilarensis* | 48,591 | Ctenomyidae |  | Octodontoidea |  |
| *C_pontifex* | 76,742 | Ctenomyidae |  | Octodontoidea |  |
| *C_porteousi* | 66,041 | Ctenomyidae |  | Octodontoidea |  |
| *C_pundti* | 117,493 | Ctenomyidae |  | Octodontoidea |  |
| *C_rionegrensis* | 92,479 | Ctenomyidae |  | Octodontoidea |  |
| *C_roigi* | 37,807 | Ctenomyidae |  | Octodontoidea | Yes |
| *C_rondoni* | 107,029 | Ctenomyidae |  | Octodontoidea |  |
| *C_rosendopascuali* | 72,270 | Ctenomyidae |  | Octodontoidea |  |
| *C_saltarius* | 82,940 | Ctenomyidae |  | Octodontoidea |  |
| *C_scagliai* | 72,263 | Ctenomyidae |  | Octodontoidea |  |
| *C_sericeus* | 34,159 | Ctenomyidae |  | Octodontoidea | Yes |
| *C_sociabilis* | 28,677 | Ctenomyidae |  | Octodontoidea | Yes |
| *C_steinbachi* | 69,476 | Ctenomyidae |  | Octodontoidea |  |
| *C_sulcidens* | 1,803,485 | Echimyidae |  | Octodontoidea |  |
| *C_talarum* | 428,201 | Ctenomyidae |  | Octodontoidea |  |
| *C_torquatus* | 235,493 | Ctenomyidae |  | Octodontoidea |  |
| *C_tuconax* | 62,420 | Ctenomyidae |  | Octodontoidea |  |
| *C_tucumanus* | 65,364 | Ctenomyidae |  | Octodontoidea |  |
| *C_tulduco* | 56,658 | Ctenomyidae |  | Octodontoidea |  |
| *C_validus* | 67,060 | Ctenomyidae |  | Octodontoidea |  |
| *C_viperinus* | 66,378 | Ctenomyidae |  | Octodontoidea |  |
| *C_yolandae* | 67,080 | Ctenomyidae |  | Octodontoidea |  |
| *Ctenomys_bicolor* | 55,566 | Ctenomyidae |  | Octodontoidea |  |
| *D_boliviensis* | 391,116 | Echimyidae |  | Octodontoidea |  |
| *D_caniceps* | 46,032 | Echimyidae |  | Octodontoidea |  |
| *D_dactylinus* | 4,382,849 | Echimyidae |  | Octodontoidea |  |
| *D_labilis* | 132,509 | Echimyidae |  | Octodontoidea |  |
| *D_peruanus* | 233,664 | Echimyidae |  | Octodontoidea |  |
| *E_chrysurus* | 1,493,625 | Echimyidae |  | Octodontoidea |  |
| *E_saturnus* | 113,286 | Echimyidae |  | Octodontoidea |  |
| *E_spinosus* | 1,071,738 | Echimyidae |  | Octodontoidea |  |
| *E_vieirai* | 94,883 | Echimyidae |  | Octodontoidea |  |
| *H_gymnurus* | 207,895 | Echimyidae |  | Octodontoidea |  |
| *I_barbarabrownae* | 26,281 | Echimyidae |  | Octodontoidea | Yes |
| *I_bistriata* | 1,844,783 | Echimyidae |  | Octodontoidea |  |
| *I_negrensis* | 609,719 | Echimyidae |  | Octodontoidea |  |
| *I_orinoci* | 146,637 | Echimyidae |  | Octodontoidea |  |
| *I_pagurus* | 245,650 | Echimyidae |  | Octodontoidea |  |
| *I_sinnamariensis* | 162,005 | Echimyidae |  | Octodontoidea |  |
| *K_amblyonyx* | 676,648 | Echimyidae |  | Octodontoidea |  |
| *L_emiliae* | 322,561 | Echimyidae |  | Octodontoidea |  |
| *M_coypus* | 2,870,255 | Echimyidae |  | Octodontoidea |  |
| *M_didelphoides* | 4,894,281 | Echimyidae |  | Octodontoidea |  |
| *M_hispidus* | 5,232,543 | Echimyidae |  | Octodontoidea |  |
| *M_leniceps* | 49,408 | Echimyidae |  | Octodontoidea |  |
| *M_macrura* | 2,597,869 | Echimyidae |  | Octodontoidea |  |
| *M_occultus* | 75,919 | Echimyidae |  | Octodontoidea |  |
| *M_stimulax* | 406,791 | Echimyidae |  | Octodontoidea |  |
| *O_albicaudus* | 161,387 | Echimyidae |  | Octodontoidea |  |
| *O_bridgesii* | 134,622 | Octodontidae |  | Octodontoidea |  |
| *O_degus* | 63,584 | Octodontidae |  | Octodontoidea |  |
| *O_edax* | 18,153 | Echimyidae |  | Octodontoidea | Yes |
| *O_gliroides* | 508,286 | Octodontidae |  | Octodontoidea |  |
| *O_lunatus* | 31,547 | Octodontidae |  | Octodontoidea | Yes |
| *O_mimax* | 123,288 | Octodontidae |  | Octodontoidea |  |
| *Octodon_pacificus* | 2,411 | Octodontidae |  | Octodontoidea | Yes |
| *P_blainvilii* | 658,028 | Echimyidae |  | Octodontoidea |  |
| *P_brasiliensis* | 53,869 | Echimyidae |  | Octodontoidea |  |
| *P_brevicauda* | 1,836,935 | Echimyidae |  | Octodontoidea |  |
| *P_canicollis* | 135,176 | Echimyidae |  | Octodontoidea |  |
| *P_carrikeri* | 191,006 | Echimyidae |  | Octodontoidea |  |
| *P_chrysaeolus* | 160,051 | Echimyidae |  | Octodontoidea |  |
| *P_cuvieri* | 3,637,416 | Echimyidae |  | Octodontoidea |  |
| *P_dasythrix* | 267,947 | Echimyidae |  | Octodontoidea |  |
| *P_decumanus* | 87,485 | Echimyidae |  | Octodontoidea |  |
| *P_echinothrix* | 1,066,447 | Echimyidae |  | Octodontoidea |  |
| *P_flavidus* | 1,350 | Echimyidae |  | Octodontoidea | Yes |
| *P_gardneri* | 493,504 | Echimyidae |  | Octodontoidea |  |
| *P_goeldii* | 1,480,204 | Echimyidae |  | Octodontoidea |  |
| *P_guairae* | 339,075 | Echimyidae |  | Octodontoidea |  |
| *P_guyannensis* | 1,913,731 | Echimyidae |  | Octodontoidea |  |
| *P_hoplomyoides* | 150,511 | Echimyidae |  | Octodontoidea |  |
| *P_kerri* | 17,607 | Echimyidae |  | Octodontoidea | Yes |
| *P_kulinae* | 175,886 | Echimyidae |  | Octodontoidea |  |
| *P_lamarum* | 444,602 | Echimyidae |  | Octodontoidea |  |
| *P_longicaudatus* | 1,609,756 | Echimyidae |  | Octodontoidea |  |
| *P_lundi* | 6,808 | Echimyidae |  | Octodontoidea | Yes |
| *P_mantiqueirensis* | 11,264 | Echimyidae |  | Octodontoidea | Yes |
| *P_medius* | 923,862 | Echimyidae |  | Octodontoidea |  |
| *P_mincae* | 19,454 | Echimyidae |  | Octodontoidea | Yes |
| *P_nigrispinus* | 426,967 | Echimyidae |  | Octodontoidea |  |
| *P_occasius* | 513,759 | Echimyidae |  | Octodontoidea |  |
| *P_oconnelli* | 55,992 | Echimyidae |  | Octodontoidea |  |
| *P_pattoni* | 585,425 | Echimyidae |  | Octodontoidea |  |
| *P_punctatus* | 59,677 | Echimyidae |  | Octodontoidea |  |
| *P_quadruplicatus* | 1,205,299 | Echimyidae |  | Octodontoidea |  |
| *P_roberti* | 2,097,851 | Echimyidae |  | Octodontoidea |  |
| *P_semispinosus* | 306,382 | Echimyidae |  | Octodontoidea |  |
| *P_semivillosus* | 78,747 | Echimyidae |  | Octodontoidea |  |
| *P_simonsi* | 2,001,152 | Echimyidae |  | Octodontoidea |  |
| *P_steerei* | 1,641,293 | Echimyidae |  | Octodontoidea |  |
| *P_sulinus* | 618,481 | Echimyidae |  | Octodontoidea |  |
| *P_thomasi* | 14,566 | Echimyidae |  | Octodontoidea | Yes |
| *P_trinitatis* | 27,643 | Echimyidae |  | Octodontoidea | Yes |
| *P_unicolor* | 21,685 | Echimyidae |  | Octodontoidea | Yes |
| *Proechimys_pattoni* | 122,341 | Echimyidae |  | Octodontoidea |  |
| *Proechimys_sp* | 57,748 | Echimyidae |  | Octodontoidea |  |
| *S_cyanus* | 103,738 | Octodontidae |  | Octodontoidea |  |
| *S_rufodorsalis* | 10,913 | Echimyidae |  | Octodontoidea | Yes |
| *T_a_albispinus* | 431,496 | Echimyidae |  | Octodontoidea |  |
| *T_a_minor* | 47,901 | Echimyidae |  | Octodontoidea |  |
| *T_apereoides* | 272,471 | Echimyidae |  | Octodontoidea |  |
| *T_barrerae* | 285,342 | Octodontidae |  | Octodontoidea |  |
| *T_dimidiatus* | 35,994 | Echimyidae |  | Octodontoidea | Yes |
| *T_eliasi* | 20,592 | Echimyidae |  | Octodontoidea | Yes |
| *T_g_bonafidei* | 10,943 | Echimyidae |  | Octodontoidea | Yes |
| *T_g_gratiosus* | 78,447 | Echimyidae |  | Octodontoidea |  |
| *T_grandis* | 371,616 | Echimyidae |  | Octodontoidea |  |
| *T_iheringi* | 29,269 | Echimyidae |  | Octodontoidea | Yes |
| *T_inermis* | 691,922 | Echimyidae |  | Octodontoidea |  |
| *T_kirchnerorum* | 5,669 | Octodontidae |  | Octodontoidea | Yes |
| *T_laurentius* | 890,396 | Echimyidae |  | Octodontoidea |  |
| *T_loschalchalerosorum* | 12,573 | Octodontidae |  | Octodontoidea | Yes |
| *T_mirapitanga* | 26,605 | Echimyidae |  | Octodontoidea | Yes |
| *T_moojeni* | 10,075 | Echimyidae |  | Octodontoidea | Yes |
| *T_pachyurus* | 937,455 | Echimyidae |  | Octodontoidea |  |
| *T_paratus* | 38,120 | Echimyidae |  | Octodontoidea | Yes |
| *T_rhipidurus* | 137,361 | Echimyidae |  | Octodontoidea |  |
| *T_s_elegans* | 139,005 | Echimyidae |  | Octodontoidea |  |
| *T_s_setosus* | 442,134 | Echimyidae |  | Octodontoidea |  |
| *T_yonenagae* | 34,220 | Echimyidae |  | Octodontoidea | Yes |
| *Thrichomys_sp* | 527,024 | Echimyidae |  | Octodontoidea |  |
| *Tympanoctomys_aureus* | 12,150 | Octodontidae |  | Octodontoidea | Yes |
